# Translational Regulation of Specific mRNAs Controls Feedback Inhibition and Survival during Macrophage Activation

**DOI:** 10.1371/journal.pgen.1004368

**Published:** 2014-06-19

**Authors:** Johanna Schott, Sonja Reitter, Janine Philipp, Katharina Haneke, Heiner Schäfer, Georg Stoecklin

**Affiliations:** 1 Helmholtz Junior Research Group Posttranscriptional Control of Gene Expression, German Cancer Research Center (DKFZ), Heidelberg, Germany, and Zentrum für Molekulare Biologie der Universität Heidelberg (ZMBH), Germany, DKFZ-ZMBH Alliance, Heidelberg, Germany; 2 Laboratory of Molecular Gastroenterology and Hepatology, Department of Internal Medicine 1, Universitätsklinikum Schleswig-Holstein Campus Kiel, Kiel, Germany; The University of Queensland, Australia

## Abstract

For a rapid induction and efficient resolution of the inflammatory response, gene expression in cells of the immune system is tightly regulated at the transcriptional and post-transcriptional level. The control of mRNA translation has emerged as an important determinant of protein levels, yet its role in macrophage activation is not well understood. We systematically analyzed the contribution of translational regulation to the early phase of the macrophage response by polysome fractionation from mouse macrophages stimulated with lipopolysaccharide (LPS). Individual mRNAs whose translation is specifically regulated during macrophage activation were identified by microarray analysis. Stimulation with LPS for 1 h caused translational activation of many feedback inhibitors of the inflammatory response including NF-κB inhibitors (*Nfkbid*, *Nfkbiz*, *Nr4a1*, *Ier3*), a p38 MAPK antagonist (*Dusp1*) and post-transcriptional suppressors of cytokine expression (*Zfp36* and *Zc3h12a*). Our analysis showed that their translation is repressed in resting and de-repressed in activated macrophages. Quantification of mRNA levels at a high temporal resolution by RNASeq allowed us to define groups with different expression patterns. Thereby, we were able to distinguish mRNAs whose translation is actively regulated from mRNAs whose polysomal shifts are due to changes in mRNA levels. Active up-regulation of translation was associated with a higher content in AU-rich elements (AREs). For one example, *Ier3* mRNA, we show that repression in resting cells as well as de-repression after stimulation depends on the ARE. Bone-marrow derived macrophages from *Ier3* knockout mice showed reduced survival upon activation, indicating that IER3 induction protects macrophages from LPS-induced cell death. Taken together, our analysis reveals that translational control during macrophage activation is important for cellular survival as well as the expression of anti-inflammatory feedback inhibitors that promote the resolution of inflammation.

## Author Summary

When macrophages encounter pathogens, they initiate inflammation by secreting pro-inflammatory factors such as the cytokine TNF. Because a prolonged or overshooting release of these factors is harmful for the organism, their production needs to be tightly controlled and shut off in due time. To ensure a rapid but transient inflammatory response, gene expression is regulated at multiple levels, including transcription, stability and translation of mRNAs. While control of transcription and mRNA stability has been studied extensively, little is known about translational regulation in macrophages. In this study, we measured the translation of all mRNAs expressed in mouse macrophages. Upon activation of macrophages with the bacterial cell wall component lipopolysaccharide, we found that many feedback inhibitors, which are important for dampening the inflammatory response, are translationally up-regulated. Translation of these mRNAs is repressed in resting cells and de-repressed after stimulation. In contrast to feedback inhibitors, most cytokines are primarily regulated by changes in mRNA abundance. Furthermore, we could show that one of the feedback inhibitors, IER3, protects macrophages from cell death during activation. Therefore, regulation at the level of translation is important for the induction of negative feedback loops and cellular survival.

## Introduction

In their function as innate immune cells, macrophages are highly sensitive to endogenous and exogenous danger signals. They sense pathogen-associated molecular patterns through Toll-like receptors (TLRs) and mount a tightly controlled immune response. The secretion of cytokines and chemokines by macrophages recruits, activates and polarizes other immune cells, while reactive oxygen species and phagocytosis directly kill microorganisms. Lipopolysaccharide (LPS), a cell wall component of gram-negative bacteria, potently activates macrophages via TLR4. Upon receptor ligation, the NF-κB pathway together with the p38 MAPK, ERK1/2 and JNK pathways causes a highly orchestrated, transient induction of numerous inflammatory genes. Such dynamic gene expression patterns are achieved by regulation at multiple levels, as exemplified by the pro-inflammatory cytokine TNF. The promoter of *Tnf* contains a cAMP responsive element and binding sites for NFAT, ETS1/ELK1, SP1, EGR proteins and NF-κB [1]. LPS also acts at the post-transcriptional level and controls the splicing, nuclear export, stability and translation of *Tnf* mRNA [2]. In their 3′ untranslated region (UTR), many cytokine mRNAs contain an AU-rich element (ARE), which recruits specific RNA-binding proteins [3]. In resting cells, TIA1, FXR1 and ZFP36 (also known as TTP) recognize the ARE and repress *Tnf* mRNA translation [4]–[6], and ZFP36 additionally causes degradation of *Tnf* mRNA [7]. Activation of the p38 MAPK pathway leads to the phosphorylation of ZFP36, whereby *Tnf* mRNA becomes partially stabilized and its translation activated [6], [8]. MicroRNAs [3] and a recently discovered stem-loop motif that acts as a constitutive RNA decay element (CDE) [9] further suppress the expression of *Tnf* and other immune-related mRNAs at the post-transcriptional level.

Not only rapid induction, but also the timely shut down of inflammatory responses is essential for immune homeostasis. The acute, excessive and systemic release of TNF, for example, can lead to septic shock, while the chronic production of pro-inflammatory cytokines sustains auto-immune diseases such as rheumatoid arthritis and Crohn's disease. In contrast, physiological immune responses induce negative feedback loops that resolve inflammation. TLR4 signaling, for example, limits itself by the induction of inhibitors that interfere with signaling complexes downstream of TLR4. Activation of the NF-κB pathway occurs via the proteasomal degradation of the NF-κB inhibitor NFKBIA (IκBα), which retains NF-κB dimers in the cytoplasm. Once in the nucleus, NF-κB dimers activate the transcription of target genes, which comprise not only cytokines but also inhibitors of NF-κB that re-export nuclear NF-κB to the cytoplasm, degrade it in the nucleus or prevent it from binding to target promoters [10], [11]. The cytoplasmic NFKBIA pool is re-filled by NF-κB-induced transcription of *Nfkbia*, whereby NF-κB activation terminates itself. In addition, rapid shut down of gene expression requires clearance of the previously synthesized mRNAs. Activation of ZFP36, for example, causes degradation of 8% of all LPS-induced mRNAs [12], and more generally the regulation of mRNA half-lives was found to strongly shape the temporal expression pattern of inflammatory genes in macrophages and dendritic cells [13], [14].

While translation of *Tnf* mRNA has been studied extensively as an individual example, the general role of translational regulation during macrophage activation remains unclear. Parallel measurements show a poor correlation between mRNA and protein abundance in many systems [15], in line with the notion that translation efficiency is a major determinant of steady-state protein levels in mouse fibroblasts [16]. So far, three studies addressed the role of translational regulation at a transcriptome-wide scale during activation of innate immune cells: In LPS-stimulated dendritic cells, mRNAs of ribosomal proteins were found to be translationally repressed, which correlated with a global drop in translation in the late phase of activation [17]. In monocytes stimulated with interferon gamma, the so-called GAIT (gamma interferon-activated inhibitor of translation) element was found to cause translational inhibition of several chemokine and chemokine receptor mRNAs [18]. Both studies focused on late stages of the response when inflammatory gene expression is shut off by negative feedback loops. A third study described translational repression of mRNAs encoding components of the mitochondrial respiratory chain early after LPS-stimulation of J774.1 cells [19]. Our goal was to investigate translational regulation early during immune activation in a more comprehensive manner by including an assessment of changes in mRNA levels.

**Figure 1:**
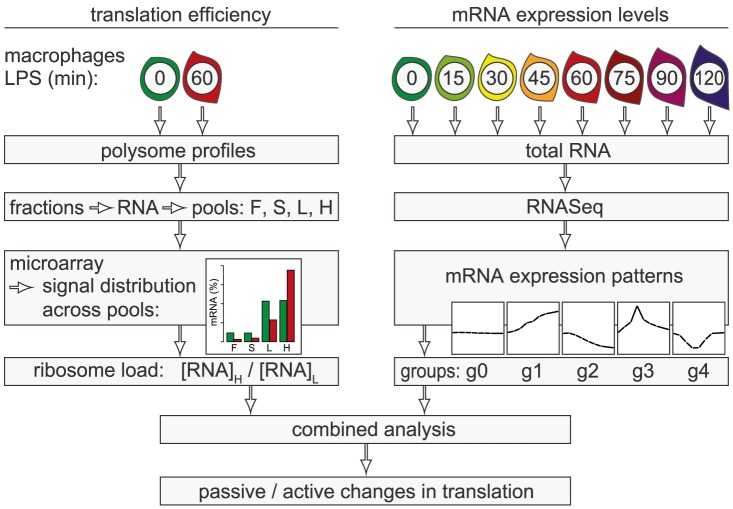
Work flow for the combined analysis of translation efficiency and mRNA expression patterns. Polysome association of mRNAs was measured by sucrose density gradient fractionation and microarray analysis of four pools (F: free, S: 40S-associated, L: light polysomes, H: heavy polysomes). For each mRNA, the distribution across the pools was calculated. The ratio H/L was used as a measure for ribosome load. mRNA levels were quantified by RNASeq at a high temporal resolution and grouped into five distinct patterns (g0–g4). By combining both data sets, mRNAs whose translation is actively regulated can be distinguished from mRNAs with passive changes in translation.

In the present study, we conducted a systematic genome-wide analysis to explore the contribution of changes in mRNA translation to the early phase of macrophage activation. To identify individual mRNAs whose translation is regulated after stimulation with LPS, we measured polysome association of mRNAs by sucrose density gradient fractionation and microarrays (Figure 1, left part of scheme). Because changes in mRNA levels are especially strong early after cell activation and can affect ribosome density without active regulation of translation efficiency, we additionally quantified mRNA levels by RNASeq at a high temporal resolution (right part of scheme). By combining both data sets, we were able to distinguish mRNAs whose translation is actively regulated from mRNAs whose translational changes are more likely to be passive. Our analysis revealed that the most frequent mode of active regulation is translation de-repression, which is prominent among inhibitors of NF-κB signaling and a factor that supports macrophage survival.

## Results

### Polysome profile analysis in activated macrophages

**Figure 2:**
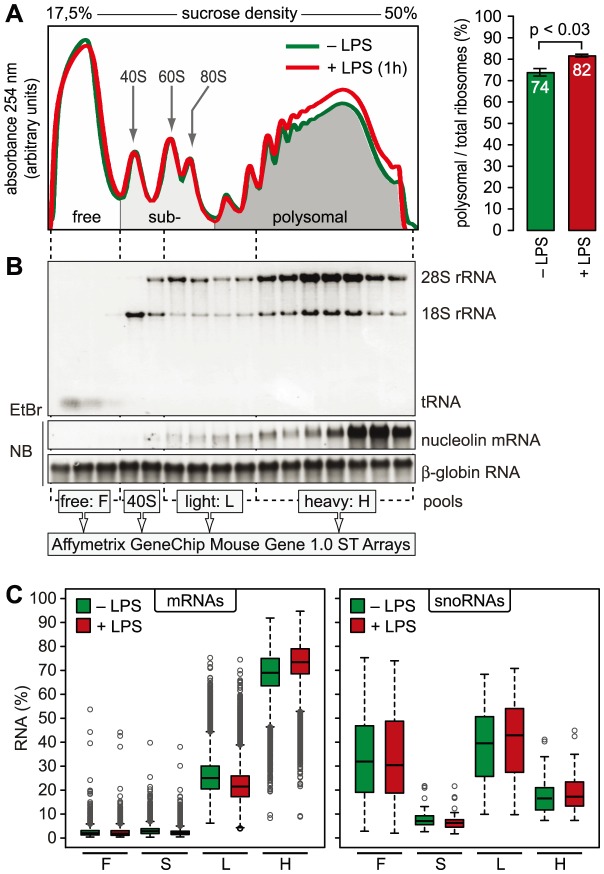
Global translation in LPS-activated macrophages. (A) Representative polysome profiles obtained by sucrose density gradient centrifugation from RAW264.7 macrophages before and after stimulation with LPS (100 ng/ml) for 1 h. Right panel: the percentage of polysomal ribosomes (mean ± SD, n = 8 for control and 3 for 1 h LPS) was calculated by quantifying the area under polysome profile curves. (B) Quality and distribution of RNA purified from 16 fractions after sucrose density gradient centrifugation. *In vitro* transcribed rabbit *HBB2* (β-globin) RNA was added as a spike-in control for equal purification efficiency; EtBr, ethidium bromide; NB, Northern blot. RNA fractions were pooled as indicated and quantified with Affymetrix Mouse Gene 1.0 ST Arrays. (C) Box plot showing the mean distribution of mRNAs and snoRNAs in the four pools as determined by microarray analysis (n = 3).

mRNAs can be separated according to the number of bound ribosomes by sucrose density gradient centrifugation, a method widely applied to monitor changes in translation initiation [20]. Polysome profiles revealed a significant increase in the percentage of polysomal ribosomes (from 74 to 82%, n = 3) after stimulation of RAW264.7 macrophages with LPS for 1 h (Figure 2A), indicating that a higher proportion of ribosomes is engaged in translation. Such a change can be due to increased initiation rates, decreased elongation rates or a reduced stoichiometric ratio of ribosomal subunits to mRNA molecules in the cell. Since we did not observe a significant change of protein synthesis by measuring [^35^S]-methionine/cysteine incorporation (Figure S1), the relative increase in polysomes is most likely not due to a general increase in the rate of translation initiation. In order to assess translation of individual mRNAs, RNA was extracted after sucrose gradient fractionation and pooled as follows: free RNA (F), 40S-associated RNA (S), RNA associated with 1–3 ribosomes (L, light polysomes) and RNA associated with >3 ribosomes (H, heavy polysomes) (Figure 2B). As a control for equal purification efficiency, we added a rabbit β-globin (*HBB2*) *in vitro* transcript (Figure 2B). The RNA pools from three biological replicates were separately hybridized to microarrays, and after annotating the probes to all mouse RNAs in the RefSeq database, the distribution of every RNA across the four pools was calculated (GEO accession number GSE52451). As expected, protein-coding RNAs (mRNAs) showed preferential association with heavy polysomes (H), whereas non-translated RNAs such as small nucleolar RNAs (snoRNAs) showed a different distribution (Figure 2C). The global up-regulation of polysome-association in LPS-activated macrophages (Figure 2A) was reflected by a shift within the polysome fractions: The median proportion of mRNAs in H increased from 69% to 73%, the median proportion in L decreased from 25% to 21% (Figure 2C).

### Translational regulation of individual mRNAs

**Figure 3:**
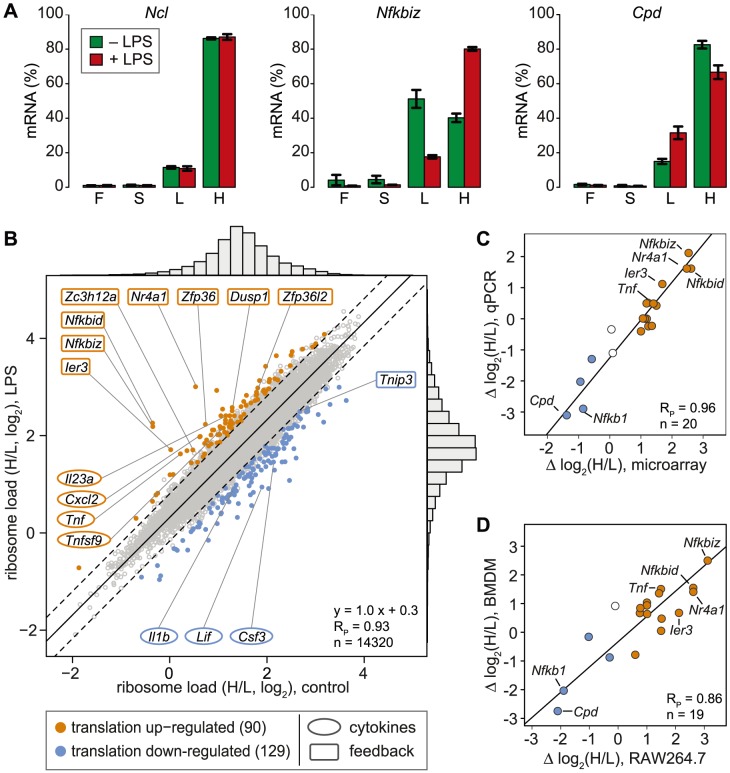
Change of translation of individual mRNAs in LPS-activated macrophages. (A) Distribution of *Ncl*, *Nfkbiz* and *Cpd* mRNA across the four pools of polysome fractions as determined by microarray analysis (mean ± SD, n = 3). (B) The ribosome load is defined as the ratio (H/L) of the proportion of an mRNA in the heavy (H) and the light (L) pool. Mean H/L was determined for each detectable, coding and RefSeq-annotated mRNA by microarray analysis (n = 3). Highlighted in orange or blue are all mRNAs that show a shift from the general trend (orthogonal regression, solid black line) beyond the cut-off of two SD (dashed lines) in ≥2/3 biological replicates and the mean; R_P_, Pearson's correlation coefficient. (C) The difference in ribosome load before and after LPS stimulation of RAW264.7 macrophages (Δ log_2_(H/L)) was determined for 20 mRNAs by microarray analysis (n = 3) and qPCR (n = 4). (D) The difference in ribosome load before and after LPS stimulation (Δ log_2_(H/L)) was determined for 19 mRNAs by qPCR in RAW264.7 cells (n = 4) and BMDM (n = 2).

In contrast to ribosome profiling, which uses sequencing of ribosome-protected fragments to obtain the density of ribosomes as a proxy for translation efficiency [21], association with different polysome fractions (F, S, L and H in our analysis) reflects the absolute number rather than the average density of ribosomes on an mRNA. A disadvantage of our method is that association with polysome fractions might not be directly proportional to the number of associated ribosomes, and that our method does not provide information on the position of ribosomes on the mRNA. On the other hand, our method preserves information on the distribution of mRNAs along the polysome profile, which is lost in ribosome profiling. Since mRNAs in general showed only minimal association with F and S (Figure 2C), we concluded that most mRNA molecules in RAW264.7 macrophages are associated with at least one ribosome. Therefore, we restricted our analysis to L and H for identification of mRNAs regulated individually at the level of translation. As a measure for the translation efficiency, we calculated for every mRNA the ratio of its proportion in H to its proportion in L (H/L), which represents a measure of ribosome load. *Ncl* mRNA, for example, has a very high ribosome load (Figure 3A, left panel and Figure 2B), and does not change its position after LPS stimulation. Some mRNAs, such as *Nfkbiz* (Figure 3A, middle panel), show a strong shift from L to H, while others, such as *Cpd* (right panel), shift in the opposite direction against the general trend of increased polysome association. By this approach, H/L was determined for 14,320 mRNAs in macrophages stimulated for 1 h with LPS and plotted against H/L in unstimulated macrophages (Figure 3B). The regression line in this plot was shifted “upwards” (y-intercept at 0.3), which reflects the general increase in polysome association after LPS stimulation. The extent to which the ribosome load of an individual mRNA deviates from the general trend corresponds to its orthogonal distance from the regression line. We chose 2 SD from the regression line as our cut-off, and asked that the mean value and at least two out of three biological replicates were outside of this cut-off. By applying these criteria, we identified 90 mRNAs that increase their ribosome load after LPS- stimulation (Figure 3B, orange dots, and Table S1), and 129 mRNAs that decrease their ribosome load after LPS stimulation (Figure 3B, blue dots, and Table S2).

For 20 mRNAs selected across the entire range of observed shifts, we confirmed the change in H/L by qPCR and obtained an excellent correlation between the qPCR and the microarray data (Pearson's correlation coefficient R_P_ = 0.96, p = 2.4×10^−11^, Figures 3C and S2). Importantly, 15 out of 19 mRNAs showed a similar shift in ribosome load in bone-marrow derived macrophages (BMDM, polysome fractionation shown in Figure S3), demonstrating that the observed regulation also occurs in primary cells (R_P_ = 0.86, p = 1.8×10^−6^, Figure 3D and S2). To our surprise, only four cytokines (out of 68 detectable in both conditions, see Table S3) were translationally up-regulated (*Tnf*, *Cxcl2*, *Il23a* and *Tnfsf9*), whereas 8 feedback inhibitors of TLR4 signaling (out of 51, see Table S4) were among the most highly up-regulated at the level of translation (Figure 3B, hypergeometric p-value for enrichment of feedback inhibitors = 9×10^−10^). NFKBID (IκBδ), NFKBIZ (IκBζ) and IER3 are reported to be direct inhibitors of RELA (p65 subunit of NF-κB) transactivation, while NR4A1 is a transcription factor for NFKBIA. DUSP1 inhibits the p38 MAPK pathway. ZFP36, ZFP36L2 (BRF1) and ZC3H12A are mRNA-binding proteins that inhibit the expression of cytokines at the post-transcriptional level. Among the translationally down-regulated mRNAs, we found one feedback inhibitor (*Tnip3*) and three cytokines (*Csf3*, *Il1b* and *Lif*).

### Correlation of translational changes with mRNA expression patterns

As suggested previously [20], [22], changes in mRNA levels can affect the average ribosome load of a transcript. When newly transcribed mRNA exits the nucleus, there is a delay until it is fully loaded with ribosomes, which leads to a transient increase of its proportion in the free or light fractions. Likewise, degradation of mRNAs is thought to occur preferentially on non-translated transcripts, which will reduce its proportion in the free or light fractions and cause a relative increase in heavy fractions [20]. Such passive shifts in ribosome load would be most prominent in the early phase of cell activation, when mRNA levels change strongly.

**Figure 4:**
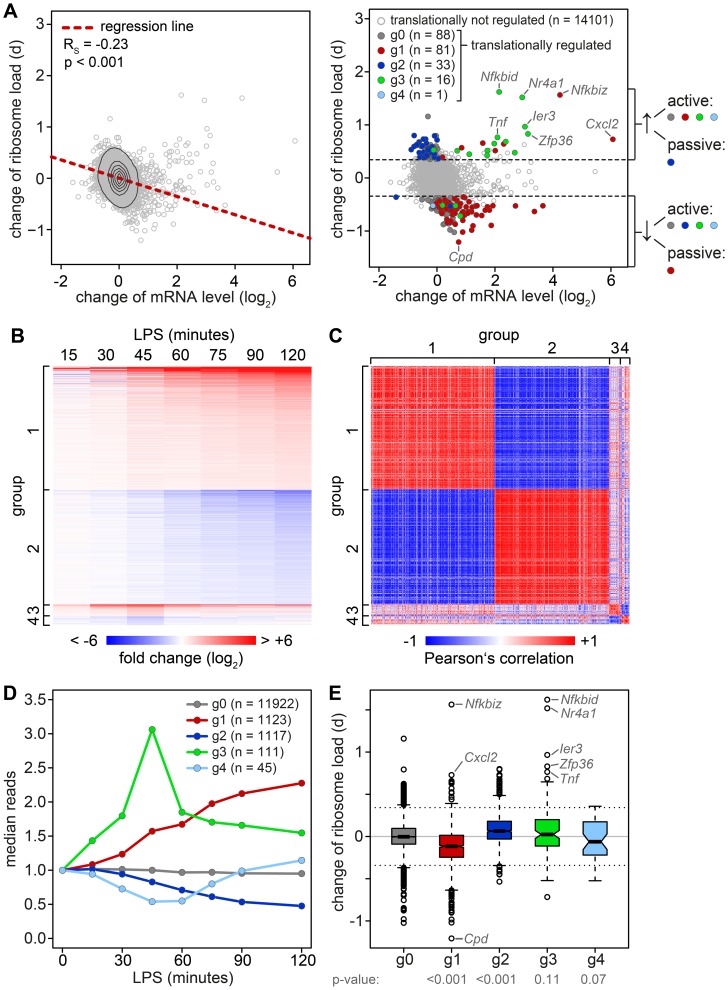
Relation between changes in mRNA levels and changes in translation. (A) Left panel: Change of translation d (orthogonal distance from the regression line in Figure 3B) versus change of mRNA levels in RAW264.7 macrophages after 1 h of LPS treatment as quantified by microarray (n = 3). Right panel: Change of translation d versus change of mRNA levels, color-coded according to groups determined by RNASeq. (B) Groups of mRNAs with different response patterns during activation of RAW264.7 macrophages, as determined by RNASeq (n = 1). g1 and g2 contain mRNAs with a first significant maximum or minimum, respectively, at or after 1 h of LPS treatment (p<0.05 and log_2_(fold change) >0.5 or <0.5). g3 and g4 contain mRNAs with one significant maximum or minimum, respectively, before 1 h. Fold changes (log_2_) to the control are represented by the intensity of blue (negative) or red (positive). (C) Pair-wise Pearson's correlation coefficient of all mRNAs in g1–4. The strength of correlation is represented by the intensity of blue (negative) or red (positive). (D) mRNA expression patterns of the groups g0–4 as median counts normalized to the median of the control condition. g0 contains all mRNAs that do not show a significant change. (E) Box plot showing the change of translation d for each of the groups; p-values were determined by two-sided Wilcoxon rank sum test.

To determine whether changes in mRNA levels are responsible for some of the shifts we observe in our ribosome load data set, we isolated total RNA at a high temporal resolution during the first 2 h of macrophage activation, and measured transcriptome-wide mRNA expression patterns by RNASeq (n = 1, GEO accession number GSE52451). Indeed, we found that the change of translation and the change of mRNA abundance show a significant negative correlation (Figure 4A, left panel, Spearman's rank correlation coefficient R_S_ = −0.23). Thus, changes in mRNA levels have to be taken into account when interpreting polysome association.

To do so, all mRNAs with a significant change in expression were divided into four groups that reflect the behavior of mRNA abundance before and immediately after 1 h of stimulation, the time point when translation was measured (Figure 4B). Within these groups, mRNA expression patterns correlate well with each other (Figure 4C). All mRNAs without a significant change in expression are in group (g)0 (Figure 4D). g1 contains 1123 mRNAs whose levels go up at the 1 h time point and reach their first significant maximum at or after 1 h of stimulation. As predicted, we found that the ribosome load of g1 mRNAs is significantly decreased by LPS stimulation compared to g0 mRNAs (p<0.001, two-sided Wilcoxon rank sum test, Figure 4E). g1 mRNAs comprise 57.4% of all mRNAs that were identified as translationally down-regulated (Table S2), but only 7.8% of all mRNAs considered in our microarray analysis. g2 contains 1117 mRNAs whose levels go down at the 1 h time point and reach their first significant minimum at or after 1 h of LPS stimulation. Similar to g1, g2 represents 34.4% of the translationally up-regulated mRNAs (Table S1), but only 7.8% of all mRNAs in our analysis. Their ribosome load is significantly increased compared to g0 mRNAs (p<0.001, Figure 4E). Hence, increasing mRNA levels (group g1) are often associated with decreasing ribosome load, while decreasing mRNA levels (group g2) are often associated with increasing ribosome load, as the shape of the bulk distribution in Figure 4A indicates. Therefore, mRNAs in g1 and g2 undergo passive shifts in ribosome load (Figure 4A, right panel).

g3 mRNAs (n = 111) show an early and transient induction of expression before 1 h of macrophage activation. As a group these mRNAs do not show a significant difference in their change of ribosome load compared to g0 (Figure 4E), yet many of the mRNAs with the strongest increase in ribosome load are part of g3 (Table S1). We concluded that the change of ribosome load of g3 mRNAs results from active translational regulation and not from passive shifts due to changes in mRNA levels. This is supported by the fact that we also observed many g3 mRNAs which do not show an increase of their ribosome load although their expression patterns are very similar to those mRNAs whose translation is strongly up-regulated (Figure S4). Similar to g3, g4 mRNAs (n = 45), which show an early and transient decrease before 1 h of LPS stimulation, do not significantly differ from g0 mRNAs in their change of ribosome load (Figure 4E).

### Active up-regulation of translation correlates with the presence of AREs

**Figure 5:**
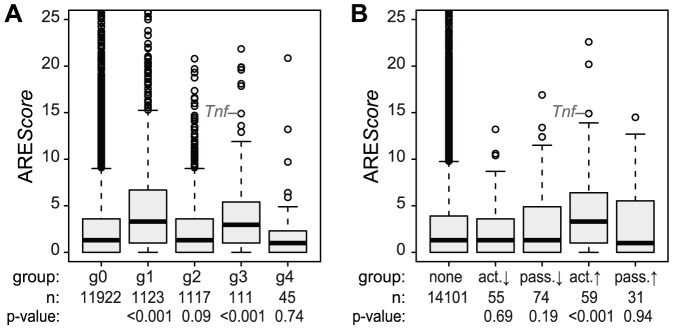
ARE scores and different patterns of regulation. (A) ARE scores were determined using the ARE*Score* algorithm and represented as boxplot for the groups g0–g4 (as defined in Figure 4); p-values were determined by two-sided Wilcoxon rank sum test. (B) Boxplot of ARE scores for groups of mRNAs with active and passive changes in ribosome load as defined in Figure 4A; p-values were determined by two-sided Wilcoxon rank sum test.

Because the ARE is often found in the 3′UTR of inflammation-related genes [3] and was shown to regulate translation of *Tnf* mRNA in LPS-stimulated macrophages [6], we looked at the frequency of AREs in the different mRNA groups described above. For this purpose, we used the ARE*Score* algorithm, which assigns a numeric value to the putative strength on an ARE [23]. As shown in Figure 5A, mRNAs whose levels increase significantly and therefore belong to group g1 or g3 (Figure 4) have significantly higher ARE*Scores* than mRNAs in g0 (p<0.001, two-sided Wilcoxon rank sum test). For translational regulation, we applied the categories as shown on the right side of Figure 4A: Translation was considered to be actively up-regulated unless the mRNA levels were decreasing (g2 mRNAs) and actively down-regulated unless the mRNA levels were increasing (g1 mRNAs). By this analysis, a significant increase in ARE*Scores* is only observed in the group of mRNAs whose translation is actively up-regulated (p<0.001, two-sided Wilcoxon rank sum test, Figure 5B).

### Translational inhibition of cytokines

**Figure 6:**
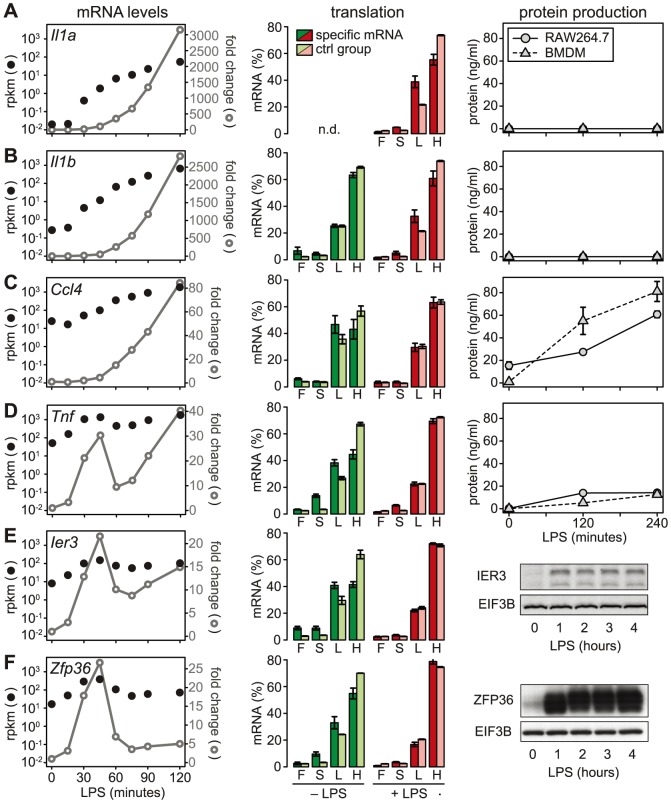
LPS-induced changes in mRNA levels, translation and protein production. (A–F) mRNA levels, polysome association and protein levels are shown for four cytokines (IL1A, IL1B, CCL4 and TNF) and two feedback inhibitors (IER3 and ZFP36). Left: mRNA levels in RAW264.7 macrophages were quantified by RNASeq (n = 1); shown are absolute expression values as reads per kilobase per million reads (rpkm) and fold change relative to the 0 minute time point. Middle: Translation in RAW264.7 macrophages was assessed by polysome fractionation and microarrays (n = 3); association of the mRNA with the free (F), 40S-bound (S), light (L) and heavy (H) pools is depicted next to the mean association of the control group of mRNAs with a similar ORF length ±25 nt. Right: Protein production by RAW264.7 macrophages or BMDM was determined by (A–D) flow cytometry (n = 3 for RAW264.7 cells, n = 4 for BMDM) or (E–F, from RAW264.7 macrophages) Western blotting (showing one representative example).

We then chose a few examples to further characterize the relationship between mRNA levels, translation and protein production. Because the ribosome load strongly correlates with the length of the open reading frame (ORF) (Figure S5), we defined for each of the selected mRNAs a control group of mRNAs with similar ORF length (±25 nt). *Il1a* mRNA, for example, has a much lower ribosome load in activated macrophages than its control group (Figure 6A, middle panel), and although the mRNA is induced >3000-fold (left panel), IL1A protein cannot be detected in the supernatant (right panel; for positive control and sensitivity of the assay see Figure S6). A similar example is *Il1b*: Its ribosome load is below that of its control group, and despite a >2500-fold induction of the mRNA during the first 2 h of LPS- stimulation, the protein is not detectably secreted into the supernatant (Figure 6B). Both IL1A and IL1B are translated as precursors that undergo proteolytic cleavage before they are secreted. LPS alone was described to prime macrophages for IL1A and IL1B production, but a second stimulus is required for cleavage of the precursors and efficient secretion [24]. In addition, it has been shown that translational repression of *Il1b* mRNA is mediated by the Janus kinase TYK2 and strongly contributes to the lack of IL1B secretion by LPS-stimulated macrophages [25]. Our data suggest that *Il1a* mRNA is subject to similar translational repression, and that the release of these cytokines in macrophages stimulated with LPS alone is prevented through a combination of translation repression and secretion.

### De-repression as a major mode of translational regulation

**Figure 7:**
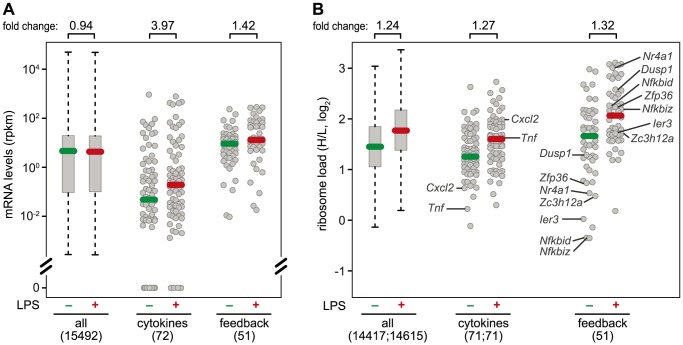
mRNA levels and translation of cytokines and feedback inhibitors. (A) Absolute mRNA expression levels were measured by RNASeq (n = 1) and depicted as rpkm before and 1 h after LPS treatment of RAW264.7 macrophages for all genes with at least one read in one of the conditions (box plot), and the subgroups of cytokines and feedback inhibitors (dot plots). Among the cytokines, 18 genes had an rpkm value of 0 before stimulation, and 4 after stimulation. (B) Ribosome load (H/L) as determined in Figure 3 for all mRNAs detectable before or 1 h after LPS treatment of RAW264.7 macrophages, separately for all genes (box plot), cytokines and feedback inhibitors (dot blots). Translationally de-repressed cytokines and feedback inhibitors are labeled.

In contrast to *Il1a* and *Il1b*, *Ccl4* mRNA is expressed at much higher levels (25 rpkm versus 0.02 rpkm for *Il1a* and 0.27 rpkm for *Il1b*), yet its translation is repressed compared to the control group (Figure 6C). After LPS stimulation, *Ccl4* mRNA is induced 80-fold and its translation is de-repressed, which is reflected by efficient secretion of CCL4 (Figure 6C). Similarly, *Tnf* mRNA levels are high (50 rpkm) in resting macrophages, and its translation is strongly suppressed (Figure 6D). Upon activation with LPS, *Tnf* mRNA shows an oscillatory induction and translation is de-repressed. TNF secretion is low but detectable in unstimulated macrophages, and induced efficiently by LPS (Figure 6D). While *Tnf* is an exception among the cytokines, five translationally regulated feedback inhibitors belong to g3 with peak expression before 1 h of LPS stimulation (Table S4). *Ier3* and *Zfp36* mRNAs, for example, show a behavior very similar to *Tnf*: Their mRNAs are well expressed in resting macrophages (8 and 17 rpkm, respectively), whereas translation is strongly repressed and the protein is barely detectable (Figure 6E and 6F). Upon LPS stimulation, mRNA levels oscillate, translation is de-repressed, and protein production is induced (Figure 6E and 6F). These examples illustrate that de-repression of translation is a frequent mode of regulation, which we observed for two cytokines and seven negative feedback inhibitors (see Figure 7).

### Different regulation of cytokines and feedback inhibitors

A systematic comparison of the 72 cytokines (including chemokines, Table S3) and 51 feedback inhibitors (Table S4) that we could track in RAW264.7 macrophages shows a distinct pattern of regulation for these two groups of genes: The majority of cytokine mRNAs is expressed at very low levels in resting macrophages (median rpkm = 4.75×10^−2^), and is strongly induced after 1 h of LPS stimulation (median rpkm = 1.89×10^−1^, 3.97-fold induction) (Figure 7A). Feedback inhibitors start with higher mRNA levels (median rpkm = 8.94), yet their increase in mRNA levels after 1 h of LPS stimulation is much weaker (median rpkm = 12.67, 1.42-fold induction). The effect of LPS on the translation of cytokines is heterogeneous: Most cytokine mRNAs follow the general trend (Figure 7B), only a few show enhanced translation (*Tnf*, *Il23a*, *Cxcl2*, *Tnfsf9*), while others are translated less efficiently (*Il1b*, *Lif*, *Csf3*) (Figure 3B). In contrast, feedback inhibitors more often show a low ribosome load in resting cells and are de-repressed upon LPS stimulation (e.g. *Ier3*, *Nfkbid*, *Nfkbiz*, *Nr4a1*, *Dusp1*, *Zfp36* and *Zc3h12a*; Figure 7B).

### The ARE of *Ier3* is required for translational regulation

**Figure 8:**
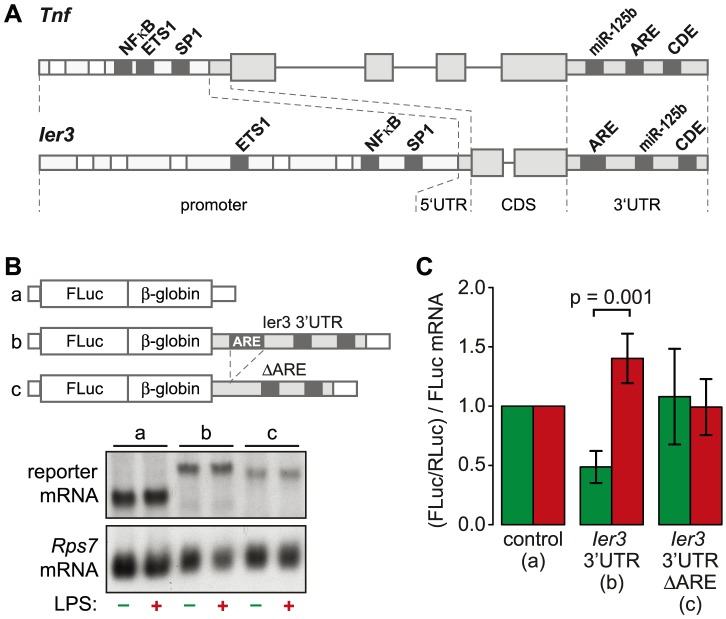
Translational regulation by the *Ier3* 3′UTR. (A) Diagram depicting the similarity of regulatory motifs between the *Ier3* and *Tnf* genes. Shared transcriptional and post-transcriptional elements are highlighted. (B) HEK-Blue mTLR4 cells were transiently co-transfected with different Firefly Luciferase reporter genes together with a Renilla Luciferase expressing plasmid. Firefly Luciferase reporter genes contained either the rabbit β-globin (*HBB2*) 3′UTR alone, the complete *Ier3* 3′UTR or the *Ier3* 3′UTR without the ARE. (C) To determine the relative translation efficiency, Firefly Luciferase (FLuc) activity was normalized to Renilla Luciferase (RLuc) activity and to the Firefly Luciferase reporter mRNA level as determined by Northern blot analysis. Bars represent mean values ± SEM (n = 4).

TNF is the most highly induced cytokine at the level of translation (Figure 3B), and its mRNA levels oscillate with a first peak during the first hour of the LPS response (Figure 6D). Several feedback inhibitors of NF-κB signaling have a very similar behavior. Out of all g3 mRNAs, *Nfkbia* and *Ier3* show the highest correlation with the expression profile of *Tnf* mRNA (R_P_ = 0.96 and 0.85, respectively). While translation of *Nfkbia* is not regulated (Table S4), *Ier3*, like *Tnf*, is translationally de-repressed after LPS stimulation (Figure 6E). Moreover, the two genes show a striking similarity of regulatory elements. The promoters of both *Tnf* and *Ier3* contain binding sites for the transcription factors NF-κB, ETS1 and SP1 (Figure 8A). In their 3′UTRs, they share three post-transcriptional regulatory elements: Both *Tnf* and *Ier3* mRNA contain a highly conserved CDE stem loop [9] and in mouse, both harbor a miR-125b binding site, which was shown to regulate the expression of mouse *Tnf* mRNA [26]; both mRNAs also contain an ARE and are validated targets of ZFP36 [7], [27]. By Luciferase assays, we confirmed that the 3′UTR of *Ier3* mediates translational regulation upon LPS stimulation. Due to the low transfection efficiency in RAW264.7 cells, we used HEK293 cells that stably express the TLR4 receptor and are therefore responsive to LPS. Because the reporter mRNAs were expressed from a heterologous MMLV promoter in HEK293 cells, their levels did not change strongly upon LPS stimulation (Figure 8B). In unstimulated cells, translation of the reporter mRNA containing the complete *Ier3* 3′UTR was suppressed more than 2-fold compared to the control reporter that contains the rabbit β-globin (*HBB2*) 3′UTR alone (Figure 8C). After stimulation with LPS, translation of the *Ier3* 3′UTR reporter was significantly increased 2.9-fold and was even more efficient than translation of the control reporter. When the ARE was deleted from the *Ier3* 3′UTR, translation was neither repressed in resting cells nor enhanced after stimulation with LPS (Figure 8C), which indicates that the ARE is involved in translational regulation of *Ier3* mRNA.

### *Ier3* protects cells from LPS-induced cell death

**Figure 9:**
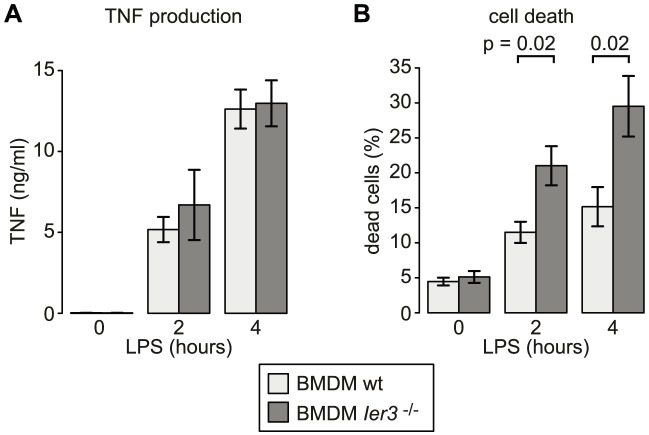
Effect of *Ier3* knockout on TNF production and survival of LPS-stimulated BMDM. (A) TNF secretion by wild type and *Ier3* knockout BMDM was measured using a bead-based flow cytometry assay. Mean values ± SEM were determined from four experiments using BMDM of two different pairs of wild type and knockout mice. (B) Cell death was measured after propidium iodide and Annexin V staining by flow cytometry. Depicted is the mean percentage of double positive cells (± SEM) based on five experiments using BMDM from three different pairs of wild type/knockout mice.

Since the transcriptional and post-transcriptional regulation of *Ier3* parallels that of *Tnf*, we speculated that IER3 may play an important role during early macrophage activation. IER3 was reported to inhibit NF-κB and limit induction of CCL2, IL6, CXCL1 and IL1B upon TLR2 stimulation [28]. Hence, we first tested whether IER3 might also affect TNF expression after TLR4 ligation. BMDM were derived from wt and *Ier3* ^−/−^ mice [29], and confirmed to be equally differentiated by the time of the experiment (Figure S7A). After LPS stimulation, both wt and *Ier3* ^−/−^ BMDM secreted similar amounts of TNF (Figure 9A), suggesting that IER3 does not affect TNF production.

IER3 was also reported to have, depending on the system, pro- or anti-apoptotic effects [30]. We therefore compared cell death in wt and *Ier3* ^−/−^ BMDM. Prior to LPS stimulation, there was no significant difference in the proportion of dead cells. After LPS stimulation, *Ier3* ^−/−^ BMDM showed twice as many dead cells as wt controls (Figure 9B). Dead BMDM were permeable for both Annexin V and propidium iodide (PI), whereas the early apoptotic population (Annexin V positive, PI negative) was barely affected by LPS stimulation (Figure S7B). We concluded that the induction of IER3 has a protective effect and contributes to the survival of macrophages during the early inflammatory response.

## Discussion

When macrophages initiate an inflammatory response, numerous secreted and intracellular proteins have to be synthesized. Despite efficient translation initiation in LPS-stimulated macrophages, the transcription of new mRNAs does not lead to an immediate increase in protein production. Rather, newly transcribed mRNAs have to compete for components of the translation machinery, and some mRNAs are loaded more efficiently with ribosomes than others. Among the 90 mRNAs that we identified as translationally up-regulated early after macrophage stimulation, 20 show a significant increase in mRNA levels (group 1 or group 3 in Table S1). For these 20, the increase in transcription and/or mRNA stability acts in synergy with enhanced translation, allowing for efficient induction of protein synthesis (Figure 4). In the subset of genes with concomitant induction of mRNA levels and translation, we find four cytokines, including *Tnf*, and two genes required for the efficient induction of *Tnf*: *Map3k8* [31] and *Dusp2* [32]. Surprisingly, the largest group of genes with concomitant induction encode negative feedback regulators of the inflammatory response (Table S4): Three direct inhibitors of RELA transactivation (*Nfkbid*, *Nfkbiz*, *Ier3*), one transcription factor for *Nfkbia* (*Nr4a1*), one phosphatase that inactivates p38 MAPK (*Dusp1*) and two RNA-binding proteins that inhibit the expression of cytokines at the post-transcriptional level (*Zfp36* and *Zc3h12a*). Four out of the seven genes (*Nfkbid*, *Dusp1*, *Zfp36* and *Zc3h12a*) are antagonists of septic shock in LPS-injected mice [33]–[36]. Translational regulation of *Nfkbid*, *Nfkbiz* and *Zc3h12a* was also described in IL1A-stimulated HeLa cells [37], suggesting that translational control observed in macrophages also operates in other cell types.

For many of the translationally up-regulated mRNAs we observe that translation is repressed in resting cells in comparison to a control group of mRNAs with similar ORF length (Figure 6). For *Tnf*, the importance of suppression at the post-transcriptional level was demonstrated by deletion of the ARE in the 3′UTR: Mice lacking the ARE in one *Tnf* allele spontaneously develop chronic inflammatory arthritis and inflammatory bowel disease [38]. In contrast to most other cytokines, *Tnf* shows comparatively high mRNA levels in resting cells (Figure 6D), which explains why suppression at the translational level is crucial. The advantage of such an expensive system is that high levels of a labile and translationally repressed mRNA prior to stimulation allow for the immediate induction of protein synthesis.

Several feedback inhibitors show a pattern similar to *Tnf*: The mRNA is already transcribed but translationally repressed in resting cells (Figure 7). Stimulation with LPS relieves translational repression and induces a transient or oscillatory induction of mRNA expression (Figure 6 and S4). Several of the translationally up-regulated cytokines and feedback inhibitors also share regulatory elements in their mRNAs: *Tnf*, *Cxcl2*, *Il23a*, *Dusp1*, *Ier3* and *Zfp36* contain known AREs [3], [39]. Indeed, by the analysis of reporter mRNA translation we could show that the ARE of *Ier3* is required for both repression of translation in unstimulated cells and de-repression upon stimulation with LPS (Figure 8). Moreover, the entire group of mRNAs that we identified as actively up-regulated at the level of translation (Figure 4A) has a significantly higher content in predicted AREs than mRNAs whose translation is not regulated (Figure 5B). This group of 59 mRNAs encodes proteins of various functions, which suggests that translational regulation mediated by AREs is involved in multiple processes besides cytokine expression and negative feedback. Notably, ZFP36, an ARE-binding protein that was shown to repress translation of *Tnf* mRNA [6], is part of this group and is therefore induced along with its targets (Figure 6F). During the first hours of the response to LPS, however, ZFP36 is strongly phosphorylated (Figure 6F, right panel) and therefore not active as a translation suppressor [6]. Nevertheless, not all mRNAs with an ARE are translationally up-regulated (Figure 5B). In fact, some cytokines that are well known to bear a strong ARE, such as *Il1b* and *Csf3*, show a decrease in ribosome load after LPS stimulation (Figure 3B and 6B). This might be due to the strong increase in mRNA levels, which is typical for cytokines (Figure 7A), or due to other regulatory mechanisms.

In addition to the ARE, CDE stem-loop motifs accelerate decay of *Tnf* mRNA in both resting and LPS-stimulated macrophages, and are also active in *Nfkbid*, *Nfkbiz* and *Ier3* mRNA [9]. Therefore, it appears that similar post-transcriptional mechanisms mediate the switch from suppression to rapid production of *Tnf* and feedback inhibitors. Interestingly, our data suggest that translational repression of feedback inhibitors in resting macrophages is just as important as inhibition of pro-inflammatory effectors like *Tnf*. Presumably, suppression of feedback inhibitors renders cells susceptible to stimulation. Immediately after stimulation, negative feedback loops are induced through translational de-repression of abundant, pre-existing mRNAs encoding feedback inhibitors, which are important for turning off the inflammatory response. In contrast to these feedback inhibitors, the induction of most cytokines strongly relies on a rapid increase in mRNA levels (Figure 7).

The highly similar expression patterns of *Tnf* and *Ier3* led us to investigate the role of *Ier3* during early macrophage activation. Besides multiple promoter elements, *Tnf* and *Ier3* share several post-transcriptional regulatory elements in their 3′UTRs (Figure 8A). Our analysis revealed that both mRNAs oscillate and are translationally de-repressed in LPS-stimulated macrophages (Figure 6). Taking both mRNA abundance and translation into account, *Ier3* shows the highest correlation with *Tnf* among all genes in our data set.

IER3 was reported to inhibit the production of the pro-inflammatory cytokines CCL2, IL6, CXCL1 and IL1B in macrophages upon TLR2 stimulation [28]. In a mouse model of inflammatory bowel disease, *Ier3* ^−/−^ mice showed an aggravated phenotype with a stronger activation of the NF-κB pathway and increased cytokine production [28]. LPS-stimulated *Ier3* ^−/−^ BMDM, however, did not produce more TNF than wt macrophages (Figure 9A). Inhibitors of NF-κB including NFKBIZ, NR4A1 and IER3 regulate not only cytokine expression but also the susceptibility to apoptosis [30], [40], [41]. Indeed we found that twice as many macrophages die after 2 and 4 h of LPS treatment when *Ier3* is deleted (Figure 9B), demonstrating that *Ier3* protects macrophages from LPS-induced cell death. In our experiments, BMDM became Annexin V-positive and permeable for PI (Figure S7B), which is indicative of necrosis rather than apoptosis.

Macrophage activation involves a complex network of pro- and anti-inflammatory signals, which ensures that inflammation is initiated, but also limited and resolved in due time. On this tightrope walk of immune homeostasis, macrophages not only have to find the right balance between activators of inflammation and negative feedback regulators, but also protect themselves from damage. While post-transcriptional regulation of cytokines has been studied extensively, our work reveals that translational regulation primarily controls feedback inhibitors.

## Materials and Methods

### Cell culture

RAW264.7 cells were cultured in Dulbecco's modified Eagle's medium (DMEM, Gibco) supplemented with 10% fetal bovine serum (FBS, Biochrome), 2 mM L-Glutamine, 100 U/ml penicillin and 0.1 mg/ml streptomycin (all PAN Biotech) at 37°C in a humidified atmosphere with 5% CO_2_. HEK293 cells stably expressing the TLR4 receptor (HEK-Blue mTLR4) were purchased from Invivogen and cultured in DMEM containing 10% FBS, 2 mM L-Glutamine, 100 U/ml penicillin, 0.1 mg/ml streptomycin, 0.1 mg/ml Normocin and 1× HEK-Blue Selection (Invivogen) at 37°C in a humidified atmosphere with 5% CO_2_. To obtain BMDM, tibia and femur of wild type or Ier3^−/−^ mice [29] were flushed with PBS. Bone-marrow cells were frozen at −80°C in FBS with 10% DMSO at a density of 1.6×10^7^ cells/ml and stored in liquid nitrogen. For differentiation of BMDM, bone-marrow cells were cultured in DMEM with 10% FBS, 2 mM L-Glutamine, 100 U/ml penicillin, 0.1 mg/ml streptomycin, 55 µM β-mercaptoethanol, 18 mM HEPES and 30% conditioned medium of L929 cells. BMDM were stained with APC anti-mouse/human ITGAM (CD11b; BLD-101211) and Alexa Fluor(R) 488 anti-mouse EMR1 (F4/80; BLD-123119) to assess differentiation by flow cytometry.

### Polysome fractionation and RNA purification

RAW264.7 macrophages or BMDM were stimulated with 100 ng/ml LPS (E. coli O111:B4, Sigma L2630) for 1 h. Ribosomes were stalled by addition of 100 µg/ml cycloheximide (CHX) for 5 min, and cells were lysed in Polysome lysis buffer (15 mM Tris-HCl pH 7.4, 15 mM MgCl_2_, 300 mM NaCl, 1% Triton-X-100, 0.1% β-mercaptoethanol, 200 U/ml RNAsin (Promega), 1 complete Mini Protease Inhibitor Tablet (Roche) per 10 ml). Nuclei were removed by centrifugation (9300× g, 4°C, 10 min) and the cytoplasmic lysate was loaded onto a sucrose density gradient (17.5–50% in 15 mM Tris-HCl pH 7.4, 15 mM MgCl_2_, 300 mM NaCl and, for fractionation from BMDM, 200 U/ml Recombinant RNAsin Ribonuclease Inhibitor, Promega). After ultracentrifugation (2.5 h, 35 000 rpm at 4°C in a SW60Ti rotor), gradients were eluted with a Teledyne Isco Foxy Jr. system into 16 fractions of similar volume. A rabbit *HBB2 in vitro* transcript was added to each fraction as a spike-in control and RNA was purified by phenol chloroform extraction. To assess RNA quality and equal purification efficiency across all fractions, the *HBB2 in vitro* transcript and endogenous *Ncl* mRNA were detected by Northern blotting.

### [^35^S]-methionine/cysteine incorporation

1.5×10^6^ RAW cells per 6 well plate were seeded 8–12 h before experiments in DMEM supplemented with 10% dialyzed FBS (PAA Laboratories), 2 mM L-Glutamine, 100 U/ml penicillin, and 0.1 mg/ml streptomycin (all PAN Biotech). Before treatment, cells were cultured in methionine- and cysteine-free medium for 1 h. Cells were treated with 100 ng/ml LPS for 1 h, and for the last 30 minutes of the treatment, 11 µCi of [^35^S]-labeled methionine and cysteine (EasyTag; PerkinElmer) was added to each well. Cells were then washed with 1× PBS, collected, and solubilized in 150 µl of lysis buffer containing 15 mM Tris, pH 7.4, 15 mM MgCl_2_, 300 mM NaCl, and 1% Triton-X-100. After centrifugation at 7800× g for 3 min, proteins were precipitated out of the supernatant by spotting 20 µl of each lysate onto Whatman paper and soaking in 5% trichloroacetic acid followed by acetone. Incorporation of ^35^S was measured in 4 ml of Ultima Gold F (PerkinElmer) using a scintillation counter (LS 6000IC; Beckman Coulter, Brea, CA). For normalization, the total protein concentration of each sample was determined using the bicinchoninic acid protein assay reagent kit (Sigma-Aldrich).

### Microarray analysis

Cytoplasmic RNA and RNA from polysome fractions was quantified with GeneChip Mouse Gene 1.0 ST Arrays. Labeling, hybridization and scanning were performed by the GeneCore Genomics Core Facility at EMBL, Heidelberg. Random primers were used for cDNA synthesis with the Ambion WT Expression Kit to avoid any bias due to poly(A) tail length. Labeling was performed with the Affymetrix GeneChip WT Terminal Labeling Kit. Probe sequences of all perfect match probes were retrieved using the Bioconductor [42] package oligo (version 1.22.0) [43]. Probes were mapped to the mouse RefSeq transcriptome as downloaded from the UCSC Genome Browser mm10 refGene table on February 5, 2013. Probes with perfect complementarity to transcripts of more than one gene (as defined by a common gene symbol) were excluded. For mapping and further processing of probe information, the R packages seqinr [44] and Biostrings [45] were used together with in-house developed Perl scripts. Expression values were quantile normalized and summarized at the gene level with the basicRMA() function of the Bioconductor package oligo and the target gene symbols as probe set names. The different pools (cytoplasmic, free, 40S-associated, light and heavy) were pre-processed as separate groups (6 samples per group), because their signal distributions might differ due to biological and not technical reasons and therefore should not be quantile normalized together. To obtain the proportion of each mRNA in a specific pool, we had to take into account how much of each pool was used for quantification. After pre-processing, the signals were corrected for the different average proportions of each pool that were used for cDNA synthesis. For example, on average 14.2% (volume) of the free RNA pool (F, control condition) was used for cDNA synthesis, but only 0.4% of the heavy polysome pool (H, control condition). The corrected signal of an individual mRNA in a specific pool was then divided by the sum of its signal in all four pools. Only protein-coding genes with at least four specific probes and well detectable expression values in the cytoplasmic samples of treated and untreated cells were included into our analysis. Pre-processed expression values and the distribution over the four pools are represented in Dataset S1.

### Definition of the group “feedback inhibitors”

Post-transcriptional inhibitors of cytokine expression and genes involved in negative feedback loops of the TLR4 response and NF-κB signaling in general were collected based on recent reviews and a PubMed search with the following terms: “TLR4” AND “negative feedback”, “LPS” AND “negative feedback”, “NF-kappaB” AND “negative feedback” and “p38” AND “negative feedback”. PubMed IDs of all sources are listed for each gene in Supplemental Table S4.

### Quantitative real-time PCR

For RT-qPCR, mRNA was reverse transcribed with random hexamer primers. Primer efficiencies were obtained from dilution curves, and a *HBB2* (rabbit β-globin) *in vitro* transcript was used for normalization in each pool of polysome fractions. The following primers (forward/reverse) were used: *HBB2* (gaaggctcatggcaagaagg/atgatgagacagcacaataaccag), *C3ar1* (tctcagtgtgcttgactgagccat/agaccaagaatgaccatggaggca), *Cpd* (tgacgtggaaggtggtatgcaaga/tcttgtcgaagctgagaagcaggt), *Csf2* (gcatgtagaggccatcaaaga/cgggtctgcacacatgtta), *Cxcl2* (aaagtttgccttgaccctgaagcc/tctttggttcttccgttgagggac), *Icosl* (tgaacttacagaccacgcctgaca/tccatcacagcccataagcagaca), *Ier3* (gagcgggccgtggtgtc/cttggcaatgttgggttcctc), *Il15ra* (agctggaacatccaccctgattga/tgtcactactgttggcactggact), *Map3k8* (aagaatggcgtgcaaactgatccc/aggacggcaccatataactcagca), *Ncl* (agggggcagaaattgatggacgat/tgggttctggggcactttg), *Nfkb1* (atgatccctacggaactgggcaaa/tgggccatctgttgacagtggtat), *Nfkbid* (atattcgtgaacataaaggcaaga/tcagtggcgttaggctctg), *Nfkbiz* (caggtgaacaccacggatt/ctcacagctcccttctggat), *Nr4a1* (tgcacagcttgggtgttgatgttc/agcaatgcgattctgcagctcttc), *Pif1* (tgactcccgagtgctgcatttcta/aggtcagaggtttgggtccatgtt), *Plk3* (ggctggcagctcgattag/gttgggagtgccacagatg), *Tk1* (tctccacacatgatcggaacacca/cagcgctgccacaattactgtctt), *Tnf* (tgcctatgtctcagcctcttc/gaggccatttgggaacttct), *Zc3h12a* (tgtgcctatcacagaccagcacat/tgaagcggtcatcatagcacacca), *Zfand2a* (tcaccctgggaggaacagaaacaa/ctgtgctgaatgcagaagttgcca) and *Zfp36* (tctcttcaccaaggccattc/atcgactggaggctctcg).

### RNASeq and data analysis

For quantification of RNA by RNASeq, RNA was purified with the EURx GeneMATRIX universal RNA purification kit, including a DNase on-column digestion. RNA libraries were prepared for sequencing using the NEBNext Ultra Directional RNA Library Prep Kit after ribosomal RNA was removed with the Ribo-Zero Magnetic Kit (Epicentre). Library preparation and sequencing was performed by the CellNetworks Deep Sequencing Core Facility at the University of Heidelberg. As spike-in controls, *in vitro* transcripts (rabbit *HBB2* and firefly luciferase) were added at a concentration of 0.4 fmol per 1 µg RNA. Reads were mapped to the mouse RefSeq transcriptome as downloaded from the UCSC Genome Browser mm10 refGene table on February 5, 2013. The sequences of the *in vitro* transcripts were included in the index. For mapping, Bowtie [46] was used allowing a maximum of two mismatches and reporting all alignments in the best stratum (settings: -a –best –stratum –v 2). With an in-house developed Perl script, read counts were summarized at the gene level discarding all reads that map to transcript isoforms of more than one gene (as defined by a common gene symbol). To calculate fold changes relative to the control condition, library size factors were estimated with the DESeq package [47].

Expression patterns were obtained as follows: A maximum was defined as a time point with a significant increase compared to the last significant minimum (or the control). Unless the maximum is the last time point, it has to be followed by time points with a smaller or not significant fold change compared to the last significant minimum (or the control), until the end of the time course or the next significant minimum is reached. A minimum was defined in an analogous way. Significance was defined as a log_2_-transformed fold change of >0.5 for maxima or <0.5 for minima and a p-value of <0.05 (see Statistical Procedures). The group g0 contains all mRNAs without any significant changes compared to the control. G1 mRNAs have the first maximum at or after 1 h of stimulation, and no minimum before the first maximum. G2 mRNAs have the first minimum at or after 1 h of stimulation, and no maximum before the first minimum. G3 mRNAs have the first maximum before 1 h of stimulation and the first minimum at or after 1 h of stimulation. G4 mRNAs have their first minimum before 1 h of stimulation and the first maximum at or after 1 h of stimulation.

Rpkm values were calculated with the following equation:
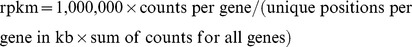



The number of 58-mers (the read length) that are unique to the transcript isoforms of one gene was obtained with an in-house developed Perl script. Read counts and the number of unique 58-mers are represented in Dataset S2.

### Plasmid construction

For plasmid MXp-GFβ (p3113), the MMLV promoter was amplified by PCR from MXh-GFP-control [48] with primers G2670/G2671 and ligated as a BamHI/KpnI fragment into the BglII/KpnI sites of pCI-puro [49]. In a second step, a GFP/β-globin fusion reporter gene was amplified with primers G2677/G2678 from pcDNA3-GFβ (p2732) [9] and ligated into the MluI/NotI sites of the first cloning product, thereby introducing an XhoI site between the MMLV promoter and GFP. For MXp-FLB (p3249, construct a in Figure 8B), GFP/β-globin was replaced with a Firefly Luciferase/β-globin fusion reporter gene, which was PCR amplified from pFLB (p2524) [50] using primers G3093/G3094 and cloned into the XhoI/EcoRI sites of MXp-GFβ (p3113). MXp-FLB-Ier3-3′UTR (p3324, construct b in Figure 8B) contains ClaI/SpeI sites that had been introduced into the BglII site at the beginning of the β-globin 3′UTR by annealing oligos G2432/G2433. These ClaI/SpeI sites were used to insert the murine *Ier3* 3′UTR after amplification by PCR with primers G2632/G2633, placing it between the β-globin ORF and the β-globin 3′UTR. To obtain MXp-FLB-Ier3-3′UTR-ΔARE (p3325) lacking the ARE (construct c in Figure 8B), the regions upstream and downstream of the ARE were amplified with primers G2632/G2680 and G2633/G2679, respectively. These two PCR products were annealed, amplified with primers G2632/G2633 and ligated into the ClaI/SpeI sites of MXp-FLB-Ier3-3′UTR (p3324).

The following primers were used:

G2670 (agtggatcccatatgggcccttccgtttc), G2671 (actcatcgattaatgcgaagagccgacgcagtctatc), G2677 (atagcggccgcgccgccatgctcgaggtgagcaagggcga), G2678 (aatacgcgttccttccgagtgagag), G3093 (attactcgaggaagacgccaaaaacataaagaaag), G3094 (ctgaggagtgaattctttgcca), G2432 (gatcctgactcatgctcagtgacgtatcgatgactactagtgtca), G2433 (gatctgacactagtagtcatcgatacgtcactgagcatgagtcag), G2632 (tgatcgataacgcgatgggtca), G2633 (gacactagtgacaggcaaatcaa), G2680 (accgaccgacacggagaaagtct), G2679 (tccgtgtcgggtcggtaagacag).

### Luciferase assay

HEK-Blue mTLR4 cells were transiently co-transfected with the MXp-FLB Firefly Luciferase Reporters and a Renilla Luciferase expressing plasmid (pCIneo-RL, p2443, [51]), and split into two wells 24 h after transfection. The cells in one well were treated with LPS (100 ng/ml, 1 h) 48 h after transfection. Cells were lysed in 300 µl of passive lysis buffer (dual-luciferase reporter assay system; Promega) per well of a 6-well dish and incubated at room temperature for 20 min. Nuclei were removed by centrifugation for 1 min at 17 000× g. 20 µl of the supernatant was mixed with 50 µl of substrate from the dual-luciferase reporter assay system, diluted 1∶3. Firefly and Renilla luciferase activities were measured on a Fluostar Optima (BMG Labtech) plate reader. In parallel, RNA was extracted and subjected to Northern blot analysis to determine the FLB reporter mRNA levels. Translation efficiency was calculated by first dividing the Firefly by the Renilla luciferase activity. This value was then normalized to the relative FLB reporter mRNA level.

### Cytokine measurement

Supernatant of stimulated BMDM was collected and stored at −80°C. Cytokine concentrations were measured with the Basic Kit mouse FlowCytomix (eBioscience) in conjunction with the mouse IL1A (BMS8611FF), IL1B (BMS8602FF), CCL4 (BMS86014FF) and the TNF (BMS860712FF) FlowCytomix Kits.

### Western blot analysis

For detection of proteins by Western blotting, cells were lysed in sample buffer (50 mM HEPES pH 7.4, 2% SDS, 10% Glycerol, 100 mM DTT). After separation on a SDS/5–20% polyacrylamide gradient gel and transfer to a 0.2 µm pore size nitrocellulose membrane (Peqlab), membranes were blocked in PBS containing 0.1% sodium azide and 5% bovine serum albumin (for IER3) or milk powder. Proteins were detected with the following antibodies diluted in PBS: ZFP36 (Carp3, abcam, ab36558-200), IER3 (Santa Cruz, sc-8454) and EIF3B (Santa Cruz, sc-16377), as well as HRP-coupled anti-goat (Santa Cruz, sc-2020) or anti-rabbit (Jackson ImmunoResearch, 711-036-152) antibody. Between the antibody incubation steps, membranes were washed in 150 mM NaCl, 50 mM Tris-HCl (pH 7.5 at 25°C), 1% Tween-20. As a luminol reagent, Western Lightning Plus-ECL Enhanced Luminol Reagent (Perkin Elmer) was used.

### Northern blot analysis

RNA was resolved by 1.2% agarose-2% formaldehyde-MOPS (morpholinepropanesulfonic acid) gel electrophoresis and blotted overnight with 8× SSC (1× SSC is 0.15 M NaCl, 0.015 M sodium citrate) onto Hybond-N+ Nylon membranes (Amersham, GE). Membranes were hybridized overnight with digoxigenin-labeled RNA probes at 55°C and washed twice with 2× SSC/0.1% SDS for 5 min and twice with 0.5× SSC/0.1% SDS for 20 min at 65°C. Alkaline phosphatase-labeled anti-DIG Fab fragments and CDP-Star substrate (both Roche) were used for detection according to the manufacturer's instructions. Templates for *in vitro* transcription of RNA probes with SP6 polymerase were obtained by PCR from cDNA of cells expressing rabbit *HBB2* (β-globin), human *Ncl* and human *Rps7* with the following primer pairs: *HBB2* (gtgcatctgtccagtg/gccgatttaggtgacactatagaataccctgaagttctc), *Ncl* (ttacaaagtcactcaggatg/gccgatttaggtgacactatagaatacttagcgtcttcg), *Rps7* (ggtggtcggaaagctatc/gccgatttaggtgacactatagaatactatagacaccag).

### Cell death analysis

Following 10 days of differentiation, BMDM were detached with non-enzymatic cell dissociation solution (SIGMA) and seeded at a density of 1.6×10^5^ cells per 12-well. After stimulation with 100 ng/ml LPS (E. coli O111:B4, Sigma L2630), cells were again detached with non-enzymatic cell dissociation solution and pelleted by centrifugation (150× g, 3 min). Cells were washed once in cold flow cytometry buffer (PBS with 0.2% FBS and 0.5 mM EDTA) and stained in 100 µl Annexin V Binding Buffer with 5 µl Alexa Fluor 647 Annexin V (BioLegend) and 5 µg/ml propdidium iodide for 15 min at room temperature. Flow cytometry measurements were performed with a BD FACSCanto II flow cytometer of the Flow Cytometry Core Facility at ZMBH, Heidelberg.

### Statistical procedures

For most statistical methods, R was used. Pearson's product moment correlation coefficients and Spearman's rank correlation coefficients were calculated with the R function cor.test(). Wilcoxon rank sum tests were performed with the function wilcox.test(). The hypergeometric p-value for enrichment was determined with phyper(). To test for differences in cell death or normalized Luciferase activity, two-sided unpaired t-tests were performed assuming equal variance. Differences in read counts of RNASeq samples were tested with the Bioconductor package DESeq [47]. DESeq estimates the dispersion that exists between biological replicates in addition to the sampling error and uses the negative binomial distribution to account for the additional variance. With the DESeq function estimateDispersions(X, method = “blind”, sharingMode = “fit-only”), we estimated the dispersion by treating the eight different samples of the LPS time course like biological replicates, assuming that the majority of genes does not change in expression. Hence, variance is rather over- than underestimated.

## Supporting Information

Figure S1 Incorporation of [^35^S]-methionine/cysteine in LPS-stimulated RAW264.7 macrophages. Protein synthesis was quantified by measuring incorporation of [^35^S]-methionine/cysteine, which was added to the culture medium 30 min after stimulation with LPS. Incorporation was measured 1 h after stimulation. Bars represent mean counts per minute (cpm) normalized to the total amount of protein (± SD, n = 3).
(JPG)

Figure S2 Confirmation of microarray results by qPCR and in BMDM. RAW264.7 macrophages or BMDM were stimulated with LPS for 1 h and mRNA was separated according to its ribosome load by sucrose density gradient centrifugation. A rabbit β-globin (*HBB2*) *in vitro* transcript was used for normalization of qPCR results in each pool. For 20 selected mRNAs, mean association with the heavy (H) fraction ± SD was determined in RAW264.7 macrophages by microarray analysis (n = 3) or qPCR (n = 4), and in BMDM by qPCR (n = 2).
(JPG)

Figure S3 Polysome fractionation from BMDM. (A) Representative polysome profiles obtained by sucrose density gradient centrifugation from BMDM before and after stimulation with LPS (100 ng/ml) for 1 h. (B) Quality and distribution of RNA purified from 11 fractions after sucrose density gradient centrifugation. *In vitro* transcribed rabbit *HBB2* RNA was added as a spike-in control for equal purification efficiency; EtBr, ethidium bromide; NB, Northern blot. RNA fractions were pooled as indicated and quantified with qPCR.
(JPG)

Figure S4 Translation of selected group 3 mRNAs. (A) Relative mRNA levels are shown for 8 g3 mRNAs whose translation is either up-regulated (orange) or unaffected/down-regulated (grey) by LPS stimulation for 1 h in RAW264.7 macrophages. (B) Association of four translationally up-regulated g3 mRNAs with the free (F), 40S-bound (S), light (L) and heavy (H) pools after polysome fractionation. Control groups as defined for Figure 6 show that translation is de-repressed by LPS treatment. (C) The same analysis was performed for four translationally unaffected or down-regulated g3 mRNAs.
(JPG)

Figure S5 Relation between ORF length and ribosome load. The box plot shows the H/L ratio for groups of mRNAs with similar ORF lengths, before and 1 h after stimulation of RAW264.7 macrophages with LPS. For each gene, the mRNA isoform with the longest ORF was used.
(JPG)

Figure S6 Standard curves of cytokines quantified with the FlowCytomix Simplex Kit. Recombinant mouse TNF, IL1A, IL1B and CCL4 were diluted as indicated and assayed with the respective mouse FlowCytomix Simplex Kits.
(JPG)

Figure S7 Differentiation and cell death of wild type (wt) and Ier3 knockout BMDM. (A) Mouse bone-marrow cells were differentiated for 10 days in the presence of 30% L929 supernatant. Expression of the differentiation markers EMR1 (F4/80; Alexa488 signal) and ITGAM (Cd11b; APC signal) was measured by flow cytometry. (B) After differentiation of mouse bone-marrow cells for 10 days in the presence of 30% L929 supernatant, cell death was measured by propidium iodide (PI) and Annexin V staining, before and 4 h after treatment with LPS (100 ng/ml).
(JPG)

Table S1 Translationally up-regulated mRNAs in 1 h LPS-stimulated RAW264.7 macrophages. The list shows all 90 mRNAs identified as translationally up-regulated by microarray analysis of polysome fractions from 1 h LPS-stimulated RAW264.7 macrophages, together with the orthogonal distance (d) from the regression line in Figure 3 as a measure of their change in polysome association, and their expression pattern as determined by RNASeq in Figure 4.
(PDF)

Table S2 Translationally down-regulated mRNAs in 1 h LPS-stimulated RAW264.7 macrophages. The list shows all 129 mRNAs identified as translationally down-regulated by microarray analysis of polysome fractions from 1 h LPS-stimulated RAW264.7 macrophages, together with the orthogonal distance (d) from the regression line in Figure 3 as a measure of their change in polysome association, and their expression pattern as determined by RNASeq in Figure 4.
(PDF)

Table S3 Cytokines and their expression features in 1 h LPS-stimulated RAW264.7 macrophages. mRNAs encoding cytokines (including chemokines) are listed, together with the orthogonal distance (d) from the regression line in Figure 3 as a measure of their change in polysome association, and their expression pattern as determined by RNASeq in Figure 4.
(PDF)

Table S4 Feedback inhibitors and their expression features in 1 h LPS-stimulated RAW264.7 macrophages. mRNAs encoding feedback inhibitors of the TLR4 response are listed, together with the orthogonal distance (d) from the regression line in Figure 3 as a measure of their change in polysome association, and their expression pattern as determined by RNASeq in Figure 4. The list of feedback inhibitors was assembled by a systematic literature search.
(PDF)

Dataset S1 Polysome association in 1 h LPS-stimulated RAW264.7 macrophages. Association of RNA with polysomes was measured by microarray analysis from polysome fractions of three biological replicates. For each protein-coding gene, pre-processed signal intensities (log_2_), calculated association with each pool of polysome fractions (F: free, S: 40S, L: light, H: heavy), the orthogonal distance from the regression line as a measure for individual regulation beyond the general trend (Fig. 3B) and the maximum ORF length are listed.
(XLSX)

Dataset S2 RNA expression patterns quantified by RNASeq in LPS-stimulated RAW264.7 macrophages. Expression of RNA was measured by RNASeq at a high temporal resolution during the first 2 h of stimulation with LPS (n = 1). For each gene, the raw read count, the expression pattern group (Fig. 4) and the number of unique 58-mers (as used for rpkm calculations) are listed.
(XLSX)
