# Supplementary figures and images for: Translational Regulation of Specific mRNAs Controls Feedback Inhibition and Survival during Macrophage Activation
Source: PLoS Genet. 2014 Jun 19;10(6):e1004368. doi: 10.1371/journal.pgen.1004368 (PMC4063670; doi:10.1371/journal.pgen.1004368)

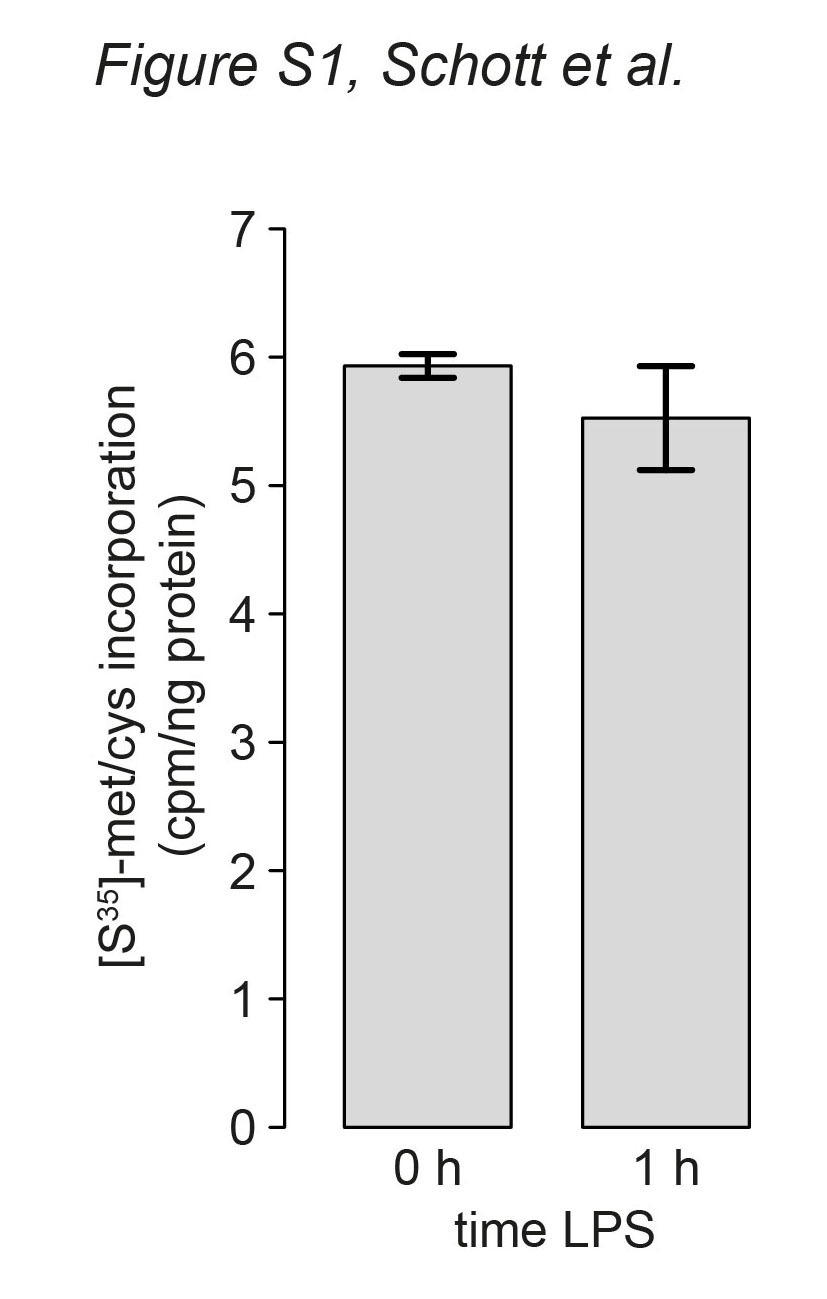

Supplement: Figure S1 — Incorporation of [35S]-methionine/cysteine in LPS-stimulated RAW264.7 macrophages. Protein synthesis was quantified by measuring incorporation of [35S]-methionine/cysteine, which was added to the culture medium 30 min after stimulation with LPS. Incorporation was measured 1 h after stimulation. Bars represent mean counts per minute (cpm) normalized to the total amount of protein (± SD, n = 3). (JPG) [file pgen.1004368.s001.jpg]

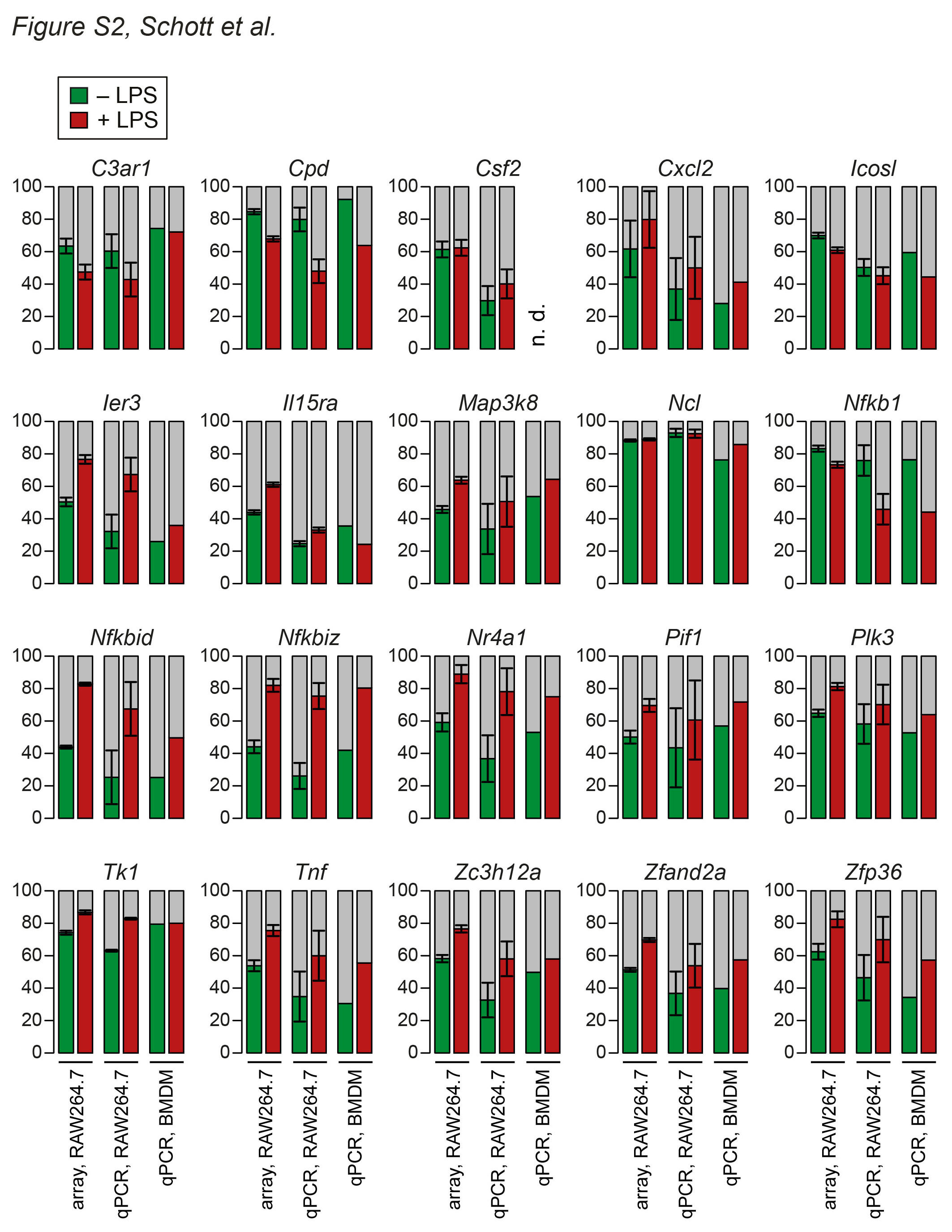

Supplement: Figure S2 — Confirmation of microarray results by qPCR and in BMDM. RAW264.7 macrophages or BMDM were stimulated with LPS for 1 h and mRNA was separated according to its ribosome load by sucrose density gradient centrifugation. A rabbit β-globin (HBB2) in vitro transcript was used for normalization of qPCR results in each pool. For 20 selected mRNAs, mean association with the heavy (H) fraction ± SD was determined in RAW264.7 macrophages by microarray analysis (n = 3) or qPCR (n = 4), and in BMDM by qPCR (n = 2). (JPG) [file pgen.1004368.s002.jpg]

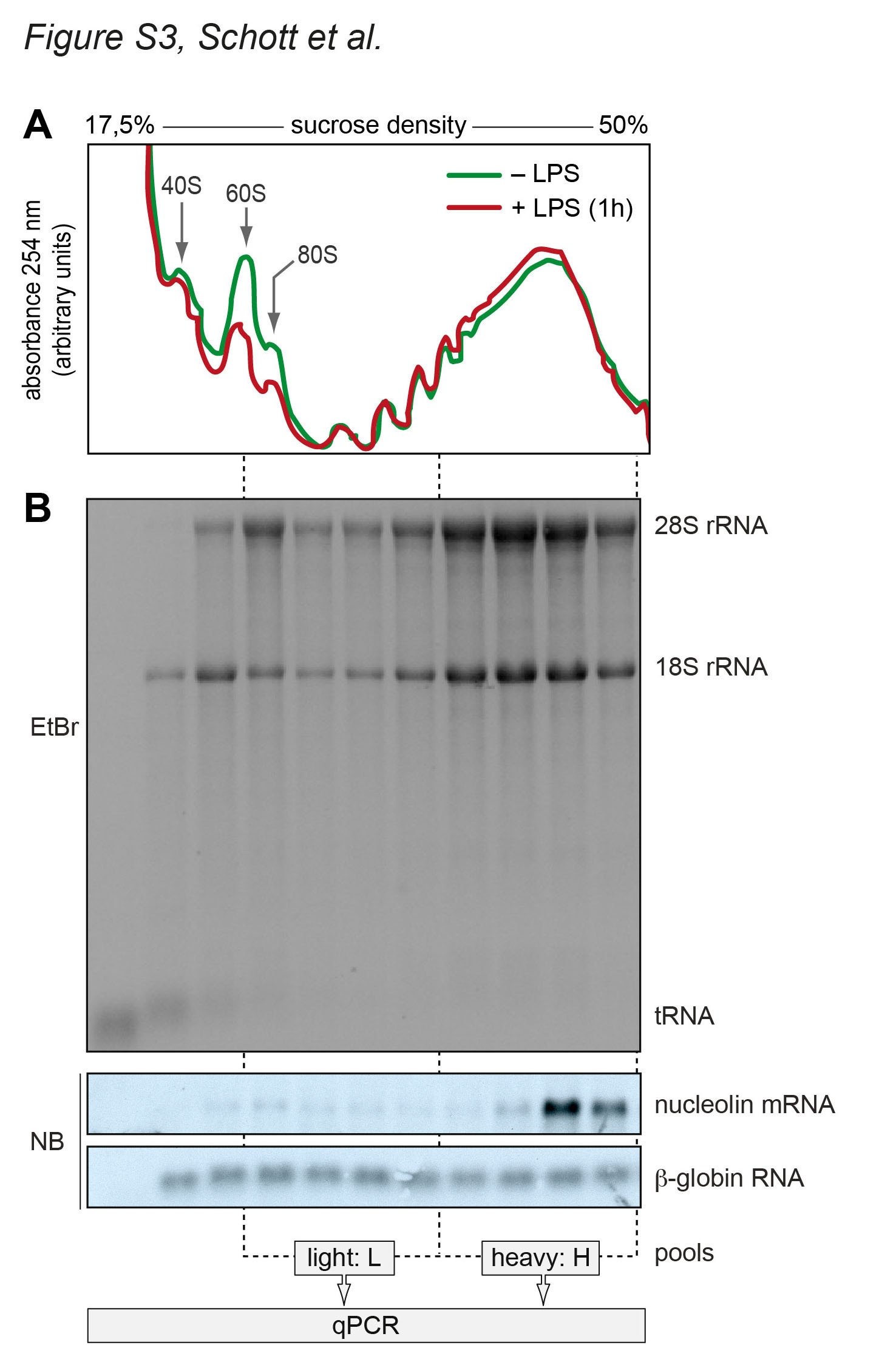

Supplement: Figure S3 — Polysome fractionation from BMDM. (A) Representative polysome profiles obtained by sucrose density gradient centrifugation from BMDM before and after stimulation with LPS (100 ng/ml) for 1 h. (B) Quality and distribution of RNA purified from 11 fractions after sucrose density gradient centrifugation. In vitro transcribed rabbit HBB2 RNA was added as a spike-in control for equal purification efficiency; EtBr, ethidium bromide; NB, Northern blot. RNA fractions were pooled as indicated and quantified with qPCR. (JPG) [file pgen.1004368.s003.jpg]

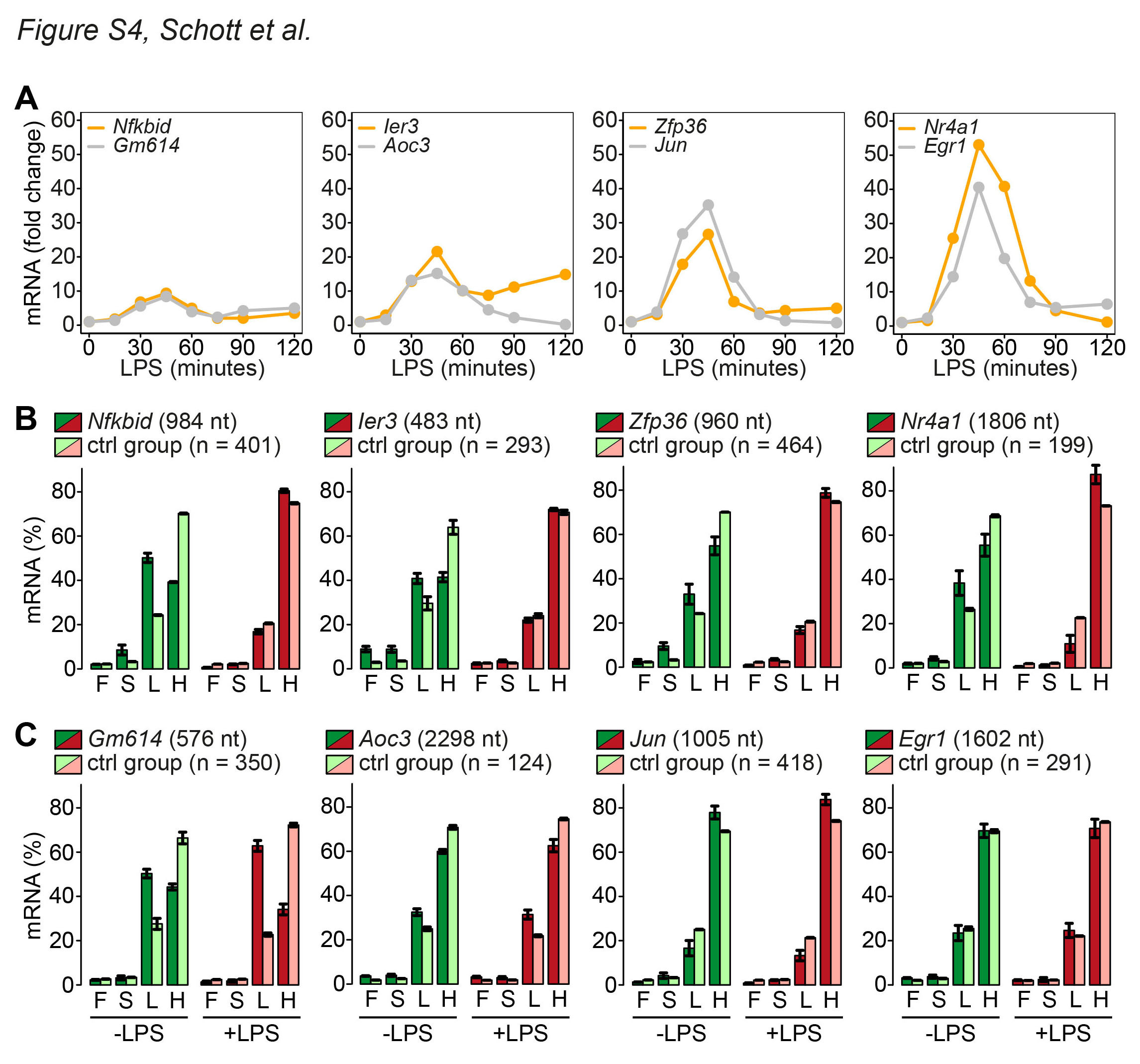

Supplement: Figure S4 — Translation of selected group 3 mRNAs. (A) Relative mRNA levels are shown for 8 g3 mRNAs whose translation is either up-regulated (orange) or unaffected/down-regulated (grey) by LPS stimulation for 1 h in RAW264.7 macrophages. (B) Association of four translationally up-regulated g3 mRNAs with the free (F), 40S-bound (S), light (L) and heavy (H) pools after polysome fractionation. Control groups as defined for Figure 6 show that translation is de-repressed by LPS treatment. (C) The same analysis was performed for four translationally unaffected or down-regulated g3 mRNAs. (JPG) [file pgen.1004368.s004.jpg]

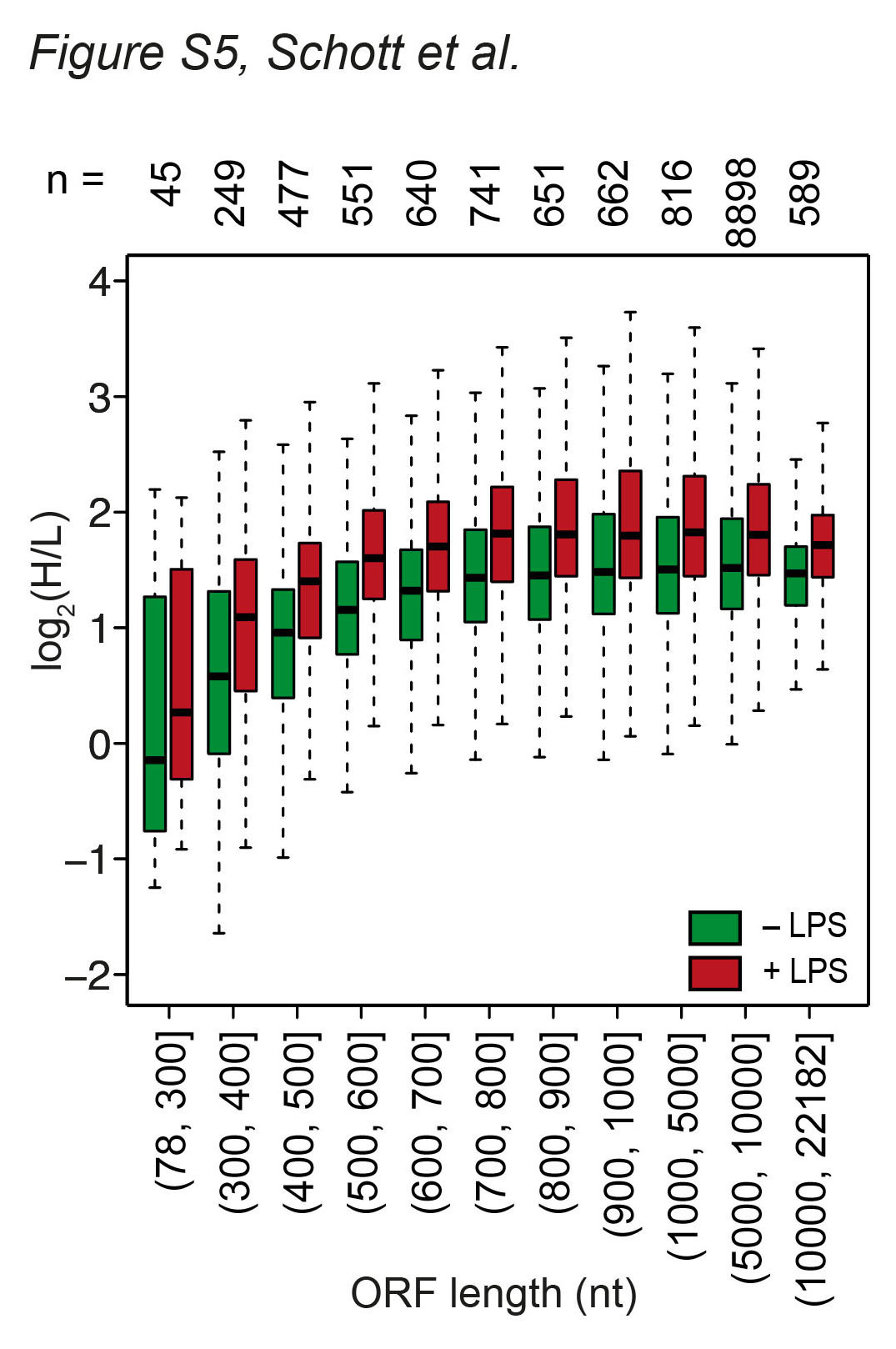

Supplement: Figure S5 — Relation between ORF length and ribosome load. The box plot shows the H/L ratio for groups of mRNAs with similar ORF lengths, before and 1 h after stimulation of RAW264.7 macrophages with LPS. For each gene, the mRNA isoform with the longest ORF was used. (JPG) [file pgen.1004368.s005.jpg]

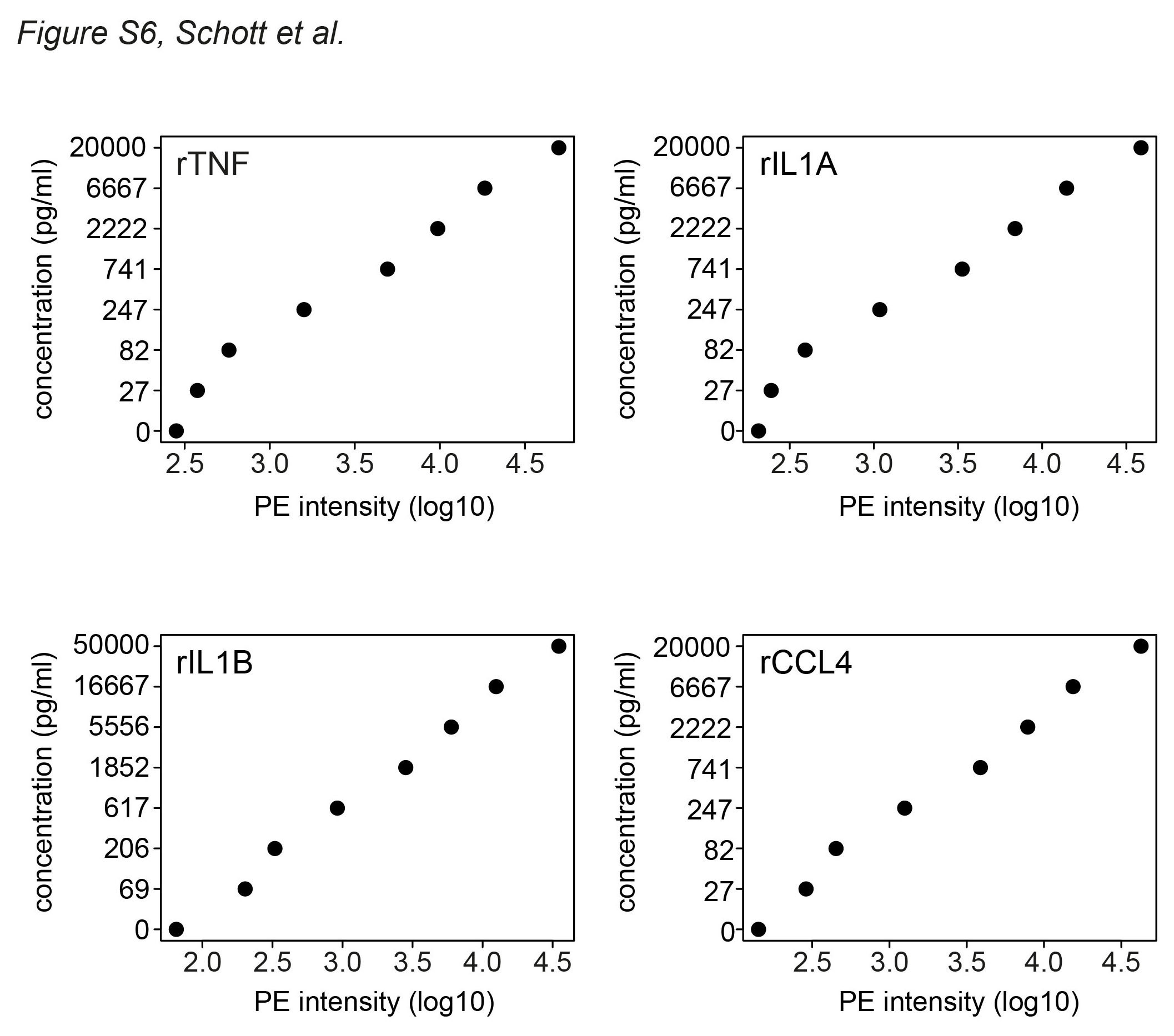

Supplement: Figure S6 — Standard curves of cytokines quantified with the FlowCytomix Simplex Kit. Recombinant mouse TNF, IL1A, IL1B and CCL4 were diluted as indicated and assayed with the respective mouse FlowCytomix Simplex Kits. (JPG) [file pgen.1004368.s006.jpg]

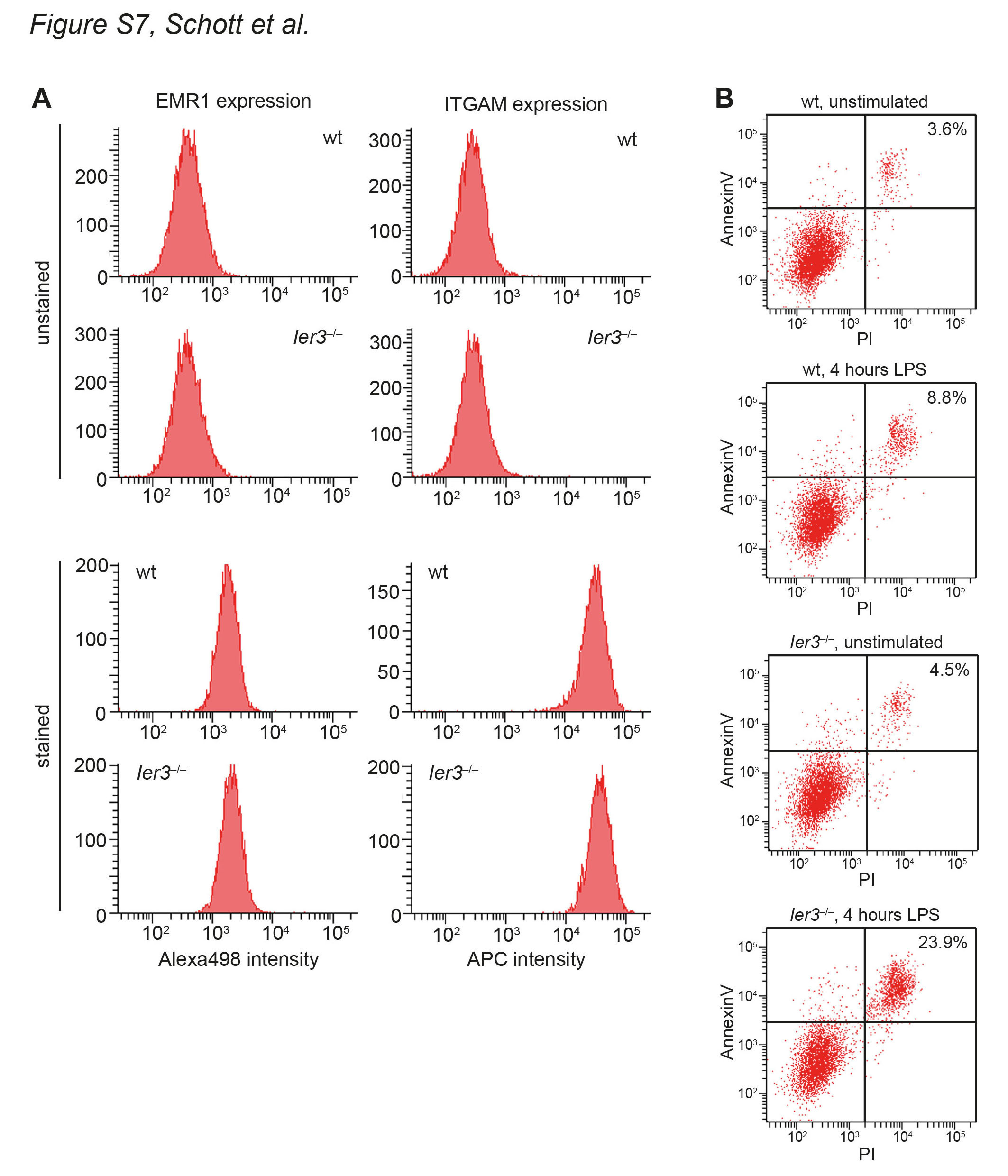

Supplement: Figure S7 — Differentiation and cell death of wild type (wt) and Ier3 knockout BMDM. (A) Mouse bone-marrow cells were differentiated for 10 days in the presence of 30% L929 supernatant. Expression of the differentiation markers EMR1 (F4/80; Alexa488 signal) and ITGAM (Cd11b; APC signal) was measured by flow cytometry. (B) After differentiation of mouse bone-marrow cells for 10 days in the presence of 30% L929 supernatant, cell death was measured by propidium iodide (PI) and Annexin V staining, before and 4 h after treatment with LPS (100 ng/ml). (JPG) [file pgen.1004368.s007.jpg]
