# Supplementary material for: Translational Regulation of Specific mRNAs Controls Feedback Inhibition and Survival during Macrophage Activation
Source: PLoS Genet. 2014 Jun 19;10(6):e1004368. doi: 10.1371/journal.pgen.1004368 (PMC4063670; doi:10.1371/journal.pgen.1004368)
Supplement: Table S1 — Translationally up-regulated mRNAs in 1 h LPS-stimulated RAW264.7 macrophages. The list shows all 90 mRNAs identified as translationally up-regulated by microarray analysis of polysome fractions from 1 h LPS-stimulated RAW264.7 macrophages, together with the orthogonal distance (d) from the regression line in Figure 3 as a measure of their change in polysome association, and their expression pattern as determined by RNASeq in Figure 4. (PDF) [file pgen.1004368.s008.pdf]

Table S1. Translationally up-regulated mRNAs in 1 h LPS-stimulated RAW264.7 macrophages.

Symbol	Name	Change of translation (d)	mRNA group*
Nfkbid	nuclear factor of kappa light polypeptide gene enhancer in B cells inhibitor, delta	1.62	3
Nfkbiz	nuclear factor of kappa light polypeptide gene enhancer in B cells inhibitor, zeta	1.56	1
Nr4a1	nuclear receptor subfamily 4, group A, member 1	1.52	3
Ugt1a6a	UDP glucuronosyltransferase 1 family, polypeptide A6A	1.16	0
Ier3	immediate early response 3	0.97	3
Zfp36	zinc finger protein 36	0.83	3
5031414D18Rik	RIKEN cDNA 5031414D18 gene	0.8	2
Impa2	inositol (myo)-1(or 4)-monophosphatase 2	0.79	0
Pank1	pantothenate kinase 1	0.79	2
Tnf	tumor necrosis factor	0.76	3
Arhgef39	Rho guanine nucleotide exchange factor (GEF) 39	0.73	2
Cxcl2	chemokine (C-X-C motif) ligand 2	0.73	1
Snx30	sorting nexin family member 30	0.73	2
Arl10	ADP-ribosylation factor-like 10	0.72	2
Crot	carnitine O-octanoyltransferase	0.68	2
Dusp1	dual specificity phosphatase 1	0.68	3
Il23a	interleukin 23, alpha subunit p19	0.66	1
Cep78	centrosomal protein 78	0.65	2
Plk3	polo-like kinase 3	0.65	3
Fam105a	family with sequence similarity 105, member A	0.64	2
Zc3h12a	zinc finger CCCH type containing 12A	0.64	1
Ppargc1b	peroxisome proliferative activated receptor, gamma, coactivator 1 beta	0.63	2
Ccnb1	cyclin B1	0.62	0
Pif1	PIF1 5'-to-3' DNA helicase homolog (S. cerevisiae)	0.61	0
Tk1	thymidine kinase 1	0.61	2
Wdr67	WD Repeat-Containing Protein 67	0.6	0
Wdyhv1	WDYHV motif containing 1	0.6	0
Abcd2	ATP-binding cassette, sub-family D (ALD), member 2	0.59	2
Fbxo48	F-box protein 48	0.59	0
Gpr155	G protein-coupled receptor 155	0.59	2
Mastl	microtubule associated serine/threonine kinase-like	0.59	2
Usp18	ubiquitin specific peptidase 18	0.59	0
Btbd8	BTB (POZ) domain containing 8	0.58	0
H2-T24	histocompatibility 2, T region locus 24	0.57	0
Zfand2a	zinc finger, AN1-type domain 2A	0.57	1
BC028528	cDNA sequence BC028528	0.56	0
Ttc5	tetratricopeptide repeat domain 5	0.56	0
Zfp36l2	zinc finger protein 36, C3H type-like 2	0.56	2
Chek2	checkpoint kinase 2	0.55	2
Mybl1	myeloblastosis oncogene-like 1	0.55	0
Hoxb6	homeobox B6	0.53	2
Klhl30	kelch-like 30	0.53	2
Map3k8	mitogen-activated protein kinase kinase kinase 8	0.53	3
Chd1l	chromodomain helicase DNA binding protein 1-like	0.52	0
Tbc1d2b	TBC1 domain family, member 2B	0.52	2
Fosb	FBJ osteosarcoma oncogene B	0.51	3

Gem	GTP binding protein (gene overexpressed in skeletal muscle)	0.51	3
Tnfsf9	tumor necrosis factor (ligand) superfamily, member 9	0.51	1
Uevld	UEV and lactate/malate dehyrogenase domains	0.51	0
Acadm	acyl-Coenzyme A dehydrogenase, medium chain	0.5	0
Nusap1	nucleolar and spindle associated protein 1	0.5	0
Slc25a40	solute carrier family 25, member 40	0.5	0
Tada2a	transcriptional adaptor 2A	0.49	2
Casp7	caspase 7	0.48	0
Clec4a3	C-type lectin domain family 4, member a3	0.48	0
Dusp2	dual specificity phosphatase 2	0.48	3
Iffo1	intermediate filament family orphan 1	0.48	2
Il15ra	interleukin 15 receptor, alpha chain	0.48	2
Mtss1	metastasis suppressor 1	0.48	0
Rhebl1	Ras homolog enriched in brain like 1	0.48	2
Hps3	Hermansky-Pudlak syndrome 3 homolog (human)	0.47	0
Prc1	protein regulator of cytokinesis 1	0.47	0
Marveld1	MARVEL (membrane-associating) domain containing 1	0.46	2
Acad12	acyl-Coenzyme A dehydrogenase family, member 12	0.45	0
Fos	FBJ osteosarcoma oncogene	0.45	3
Racgap1	Rac GTPase-activating protein 1	0.45	0
Sgol2	shugoshin-like 2 (S. pombe)	0.45	0
Sgsh	N-sulfoglucosamine sulfohydrolase (sulfamidase)	0.45	2
Abcd1	ATP-binding cassette, sub-family D (ALD), member 1	0.44	0
Fgd4	FYVE, RhoGEF and PH domain containing 4	0.44	2
Junb	Jun-B oncogene	0.43	3
Ptk2b	PTK2 protein tyrosine kinase 2 beta	0.42	0
Rasa3	RAS p21 protein activator 3	0.42	2
Sipa1	signal-induced proliferation associated gene 1	0.42	2
Slco2b1	solute carrier organic anion transporter family, member 2b1	0.42	0
Bora	bora, aurora kinase A activator	0.41	0
Inpp5d	inositol polyphosphate-5-phosphatase D	0.41	0
Ttk	Ttk protein kinase	0.41	0
Alox5	arachidonate 5-lipoxygenase	0.4	0
Aurkb	aurora kinase B	0.39	0
Fbxo9	f-box protein 9	0.39	0
Kbtbd11	kelch repeat and BTB (POZ) domain containing 11	0.39	2
Kif20a	kinesin family member 20A	0.39	0
Pus10	pseudouridylate synthase 10	0.39	0
Rgs14	regulator of G-protein signaling 14	0.39	2
Traf3ip3	TRAF3 interacting protein 3	0.39	2
Troap	trophinin associated protein	0.39	1
Accs	1-aminocyclopropane-1-carboxylate synthase (non-functional)	0.36	2
Cep55	centrosomal protein 55	0.36	0
Appl2	adaptor protein, phosphotyrosine interaction, PH domain and leucine zipper containing 2	0.35	0

* Group 0, all mRNAs with no significant change in expression levels during 2h stimulation of RAW264.7 macrophages with LPS; Group 1, mRNAs with a significant maximum at or after 1h; Group 2, mRNAs with a significant minimum at or after 1h; Group 3, mRNAs with a significant maximum before 1h; Group 4, mRNAs with a significant minimum before 1h.
