# Supplementary material for: Translational Regulation of Specific mRNAs Controls Feedback Inhibition and Survival during Macrophage Activation
Source: PLoS Genet. 2014 Jun 19;10(6):e1004368. doi: 10.1371/journal.pgen.1004368 (PMC4063670; doi:10.1371/journal.pgen.1004368)
Supplement: Table S2 — Translationally down-regulated mRNAs in 1 h LPS-stimulated RAW264.7 macrophages. The list shows all 129 mRNAs identified as translationally down-regulated by microarray analysis of polysome fractions from 1 h LPS-stimulated RAW264.7 macrophages, together with the orthogonal distance (d) from the regression line in Figure 3 as a measure of their change in polysome association, and their expression pattern as determined by RNASeq in Figure 4. (PDF) [file pgen.1004368.s009.pdf]

Table S2. Translationally down-regulated mRNAs in 1 h LPS-stimulated RAW264.7 macrophages.

Symbol	Name	Change of translation (d)	mRNA group*
Cpd	carboxypeptidase D	-1.21	1
Agpat4	1-acylglycerol-3-phosphate O-acyltransferase 4 (lysophosphatidic acid acyltransferase, delta)	-1.02	1
Dhx40	DEAH (Asp-Glu-Ala-His) box polypeptide 40	-1.02	0
Emr1	EGF-like module containing, mucin-like, hormone receptor-like sequence 1	-0.98	0
Kcnd1	potassium voltage-gated channel, Shal-related family, member 1	-0.98	1
Lif	leukemia inhibitory factor	-0.9	1
C3ar1	complement component 3a receptor 1	-0.89	1
Anxa5	annexin A5	-0.88	0
C3	complement component 3	-0.87	1
Asb2	ankyrin repeat and SOCS box-containing 2	-0.86	0
Plin2	perilipin 2	-0.86	0
Sra1	steroid receptor RNA activator 1	-0.85	0
Pdlim5	PDZ and LIM domain 5	-0.84	1
Nfkb1	nuclear factor of kappa light polypeptide gene enhancer in B cells 1, p105	-0.83	1
Swap70	SWA-70 protein	-0.81	1
Ptpn23	protein tyrosine phosphatase, non-receptor type 23	-0.8	1
Lcat	lecithin cholesterol acyltransferase	-0.78	1
Hist2h3c2	histone cluster 2, H3c2	-0.75	0
Aoc2	amine oxidase, copper containing 2 (retina-specific)	-0.74	1
Gm614	predicted gene 614	-0.72	3
Csf3	colony stimulating factor 3 (granulocyte)	-0.71	1
Abcg4	ATP-binding cassette, sub-family G (WHITE), member 4	-0.7	1
Pdlim7	PDZ and LIM domain 7	-0.7	1
Pilrb1	paired immunoglobin-like type 2 receptor beta 1	-0.7	0
Sqstm1	sequestosome 1	-0.7	1
Creb5	cAMP responsive element binding protein 5	-0.69	1
Atg2a	autophagy related 2A	-0.68	0
Tlr9	toll-like receptor 9	-0.68	0
Stap1	signal transducing adaptor family member 1	-0.67	1
Tnfaip2	tumor necrosis factor, alpha-induced protein 2	-0.67	1
Wdr1	WD repeat domain 1	-0.67	1
Nfkb2	nuclear factor of kappa light polypeptide gene enhancer in B cells 2, p49/p100	-0.65	1
Ehd1	EH-domain containing 1	-0.64	1
Icosl	icos ligand	-0.64	1
Rab11fip1	RAB11 family interacting protein 1 (class I)	-0.64	1
Rhbdd1	rhomboid domain containing 1	-0.64	1
H2-K1	histocompatibility 2, K1, K region	-0.63	0
Irg1	immunoresponsive gene 1	-0.63	1
Mpeg1	macrophage expressed gene 1	-0.63	0
Ap3b1	adaptor-related protein complex 3, beta 1 subunit	-0.62	0
Ubox5	U box domain containing 5	-0.62	0
Dusp4	dual specificity phosphatase 4	-0.61	1
Slfn2	schlafen 2	-0.61	1
Tnfrsf1b	tumor necrosis factor receptor superfamily, member 1b	-0.61	1
Plek	pleckstrin	-0.6	1
Cdk18	cyclin-dependent kinase 18	-0.59	1

Gpatch2l	G patch domain containing 2 like	-0.59	0
Slc31a2	solute carrier family 31, member 2	-0.59	1
Ap2b1	adaptor-related protein complex 2, beta 1 subunit	-0.58	0
Glul	glutamate-ammonia ligase (glutamine synthetase)	-0.58	0
Gm11127	predicted gene 11127	-0.58	0
Osbpl8	oxysterol binding protein-like 8	-0.58	1
Gm15080	predicted gene 15080	-0.57	0
Pus3	pseudouridine synthase 3	-0.57	1
Slc11a2	solute carrier family 11 (proton-coupled divalent metal ion transporters), member 2	-0.57	1
Bcl6b	B cell CLL/lymphoma 6, member B	-0.56	1
Gm7609	predicted pseudogene 7609	-0.56	0
Pik3r5	phosphoinositide-3-kinase, regulatory subunit 5, p101	-0.56	1
Apbb3	amyloid beta (A4) precursor protein-binding, family B, member 3	-0.55	1
Arl5c	ADP-ribosylation factor-like 5C	-0.55	1
Cd40	CD40 antigen	-0.55	1
Dcpp1	demilune cell and parotid protein 1	-0.55	0
Gigyf2	GRB10 interacting GYF protein 2	-0.55	1
Tm9sf4	transmembrane 9 superfamily protein member 4	-0.55	1
Ets2	E26 avian leukemia oncogene 2, 3' domain	-0.54	1
Itga5	integrin alpha 5 (fibronectin receptor alpha)	-0.54	1
Kif4	kinesin family member 4	-0.54	0
Por	P450 (cytochrome) oxidoreductase	-0.54	1
Spag9	sperm associated antigen 9	-0.54	1
Trim13	tripartite motif-containing 13	-0.54	1
Cd69	CD69 antigen	-0.53	1
Fam71f2	family with sequence similarity 71, member F2	-0.53	2
Gadd45a	growth arrest and DNA-damage-inducible 45 alpha	-0.53	1
Rasip1	Ras interacting protein 1	-0.53	1
Ythdc2	YTH domain containing 2	-0.53	0
2310014L17 Rik	RIKEN cDNA 2310014L17 gene	-0.52	3
Cybb	cytochrome b-245, beta polypeptide	-0.52	1
Ddah2	dimethylarginine dimethylaminohydrolase 2	-0.52	1
Eftud1	elongation factor Tu GTP binding domain containing 1	-0.52	0
Erbb2ip	Erbb2 interacting protein	-0.52	1
Il1b	interleukin 1 beta	-0.52	1
Kcnj2	potassium inwardly-rectifying channel, subfamily J, member 2	-0.52	4
Lpcat2	lysophosphatidylcholine acyltransferase 2	-0.52	0
Niacr1	niacin receptor 1	-0.52	1
Nos2	nitric oxide synthase 2, inducible	-0.52	1
Star	steroidogenic acute regulatory protein	-0.52	3
Traf1	TNF receptor-associated factor 1	-0.52	1
Cdo1	cysteine dioxygenase 1, cytosolic	-0.51	1
Eya3	eyes absent 3 homolog (Drosophila)	-0.51	0
Tank	TRAF family member-associated Nf-kappa B activator	-0.51	1
Dcstamp	dentrocyte expressed seven transmembrane protein	-0.5	1
Hivep3	human immunodeficiency virus type I enhancer binding protein 3	-0.5	1
Necab3	N-terminal EF-hand calcium binding protein 3	-0.5	0
Uba6	ubiquitin-like modifier activating enzyme 6	-0.5	1
Hgsnat	heparan-alpha-glucosaminide N-acetyltransferase	-0.49	0

Kctd12	potassium channel tetramerisation domain containing 12	-0.49	1
Pla2g4c	phospholipase A2, group IVC (cytosolic, calcium-independent)	-0.49	1
Atp1a1	ATPase, Na+/K+ transporting, alpha 1 polypeptide	-0.48	0
Fam129b	family with sequence similarity 129, member B	-0.48	0
H2-Q4	histocompatibility 2, Q region locus 4	-0.48	1
Sod2	superoxide dismutase 2, mitochondrial	-0.48	1
Sfr1	SWI5 dependent recombination repair 1	-0.47	0
Gm4636	predicted gene 4636	-0.46	0
Gripap1	GRIP1 associated protein 1	-0.46	1
Il2rg	interleukin 2 receptor, gamma chain	-0.46	0
Rhoc	ras homolog gene family, member C	-0.45	1
Sort1	sortilin 1	-0.45	0
Itpkb	inositol 1,4,5-trisphosphate 3-kinase B	-0.44	1
Ngly1	N-glycanase 1	-0.44	0
Slfn9	schlafen 9	-0.44	0
Tgfb1i1	transforming growth factor beta 1 induced transcript 1	-0.44	0
Xrn2	5'-3' exoribonuclease 2	-0.44	0
Abcb1b	ATP-binding cassette, sub-family B (MDR/TAP), member 1B	-0.43	0
Pold2	polymerase (DNA directed), delta 2, regulatory subunit	-0.43	0
Mtmr7	myotubularin related protein 7	-0.42	1
Zswim1	zinc finger SWIM-type containing 1	-0.42	0
1810026J23 Rik	RIKEN cDNA 1810026J23 gene	-0.41	0
B3galt4	UDP-Gal:betaGlcNAc beta 1,3-galactosyltransferase, polypeptide 4	-0.41	0
Ccdc15	coiled-coil domain containing 15	-0.41	1
Sdcbp	syndecan binding protein	-0.41	0
Taf1	TAF1 RNA polymerase II, TATA box binding protein (TBP)-associated factor	-0.41	0
Ugt1a5	UDP glucuronosyltransferase 1 family, polypeptide A5	-0.41	0
Smox	spermine oxidase	-0.39	1
Ccdc25	coiled-coil domain containing 25	-0.38	0
Mknk2	MAP kinase-interacting serine/threonine kinase 2	-0.38	0
Olfr1162	olfactory receptor 1162	-0.38	0
Dcbld2	discoidin, CUB and LCCL domain containing 2	-0.37	1
Cnr2	cannabinoid receptor 2 (macrophage)	-0.36	2
Tnip3	TNFAIP3 interacting protein 3	-0.36	1

* Group 0, all mRNAs with no significant change in expression levels during 2h stimulation of RAW264.7 macrophages with LPS; Group 1, mRNAs with a significant maximum at or after 1h; Group 2, mRNAs with a significant minimum at or after 1h; Group 3, mRNAs with a significant maximum before 1h; Group 4, mRNAs with a significant minimum before 1h.
