# Supplementary material for: Translational Regulation of Specific mRNAs Controls Feedback Inhibition and Survival during Macrophage Activation
Source: PLoS Genet. 2014 Jun 19;10(6):e1004368. doi: 10.1371/journal.pgen.1004368 (PMC4063670; doi:10.1371/journal.pgen.1004368)
Supplement: Table S3 — Cytokines and their expression features in 1 h LPS-stimulated RAW264.7 macrophages. mRNAs encoding cytokines (including chemokines) are listed, together with the orthogonal distance (d) from the regression line in Figure 3 as a measure of their change in polysome association, and their expression pattern as determined by RNASeq in Figure 4. (PDF) [file pgen.1004368.s010.pdf]

Table S3. Cytokines and their expression features in 1 h LPS-stimulated RAW264.7 macrophages.

Symbol	Name	Change of translation (d)	mRNA group*
Ccl1	chemokine (C-C motif) ligand 1	-0.12	0
Ccl11	chemokine (C-C motif) ligand 11	-0.04	0
Ccl12	chemokine (C-C motif) ligand 12	n.d.	n.d.
Ccl17	chemokine (C-C motif) ligand 17	-0.2	0
Ccl19	chemokine (C-C motif) ligand 19	-0.23	0
Ccl2	chemokine (C-C motif) ligand 2	-0.43	1
Ccl20	chemokine (C-C motif) ligand 20	0	1
Ccl22	chemokine (C-C motif) ligand 22	-0.19	1
Ccl24	chemokine (C-C motif) ligand 24	-0.22	0
Ccl25	chemokine (C-C motif) ligand 25	0.09	0
Ccl26	chemokine (C-C motif) ligand 26	n.d.	n.d.
Ccl27a	chemokine (C-C motif) ligand 27A	-0.11	0
Ccl28	chemokine (C-C motif) ligand 28	n.d.	n.d.
Ccl3	chemokine (C-C motif) ligand 3	0.32	1
Ccl4	chemokine (C-C motif) ligand 4	0.63	1
Ccl5	chemokine (C-C motif) ligand 5	-0.34	1
Ccl6	chemokine (C-C motif) ligand 6	-0.04	0
Ccl7	chemokine (C-C motif) ligand 7	-0.14	1
Ccl8	chemokine (C-C motif) ligand 8	n.d.	n.d.
Ccl9	chemokine (C-C motif) ligand 9	0.01	1
Cd40lg	CD40 ligand	n.d.	n.d.
Cd70	CD70 antigen	n.d.	n.d.
Csf1	colony stimulating factor 1 (macrophage)	-0.57	1
Csf2	colony stimulating factor 2 (granulocyte-macrophage)	-0.19	1
Csf3	colony stimulating factor 3 (granulocyte)	-0.71	1
Cxcl1	chemokine (C-X-C motif) ligand 1	0.07	1
Cxcl10	chemokine (C-X-C motif) ligand 10	0.12	1
Cxcl11	chemokine (C-X-C motif) ligand 11	-0.02	1
Cxcl12	chemokine (C-X-C motif) ligand 12	0.02	0
Cxcl13	chemokine (C-X-C motif) ligand 13	n.d.	n.d.
Cxcl14	chemokine (C-X-C motif) ligand 14	0.27	0
Cxcl15	chemokine (C-X-C motif) ligand 15	n.d.	n.d.
Cxcl16	chemokine (C-X-C motif) ligand 16	n.d.	n.d.
Cxcl17	chemokine (C-X-C motif) ligand 17	-0.09	0
Cxcl2	chemokine (C-X-C motif) ligand 2	0.73	1
Cxcl3	chemokine (C-X-C motif) ligand 3	0.08	1
Cxcl5	chemokine (C-X-C motif) ligand 5	n.d.	n.d.
Cxcl9	chemokine (C-X-C motif) ligand 9	n.d.	n.d.
Fasl	Fas ligand (TNF superfamily, member 6)	n.d.	n.d.
Ifna1	interferon alpha 1	0.25	0
Ifna11	interferon alpha 11	n.d.	n.d.
Ifna12	interferon alpha 12	n.d.	n.d.
Ifna13	interferon alpha 13	n.d.	n.d.
Ifna14	interferon, alpha 14	-0.17	0
Ifna2	interferon alpha 2	-0.14	0
Ifna4	interferon alpha 4	n.d.	n.d.

Ifna5	interferon alpha 5	-0.15	0
Ifna6	interferon alpha 6	n.d.	n.d.
Ifna7	interferon alpha 7	n.d.	n.d.
Ifna9	interferon alpha 9	0.03	0
Ifnab	interferon alpha B	0.23	0
Ifnb1	interferon beta 1, fibroblast	n.d.	n.d.
Ifne	interferon epsilon	-0.16	0
Ifng	interferon gamma	-0.07	0
Ifnk	interferon kappa	n.d.	n.d.
Ifnl2	interferon lambda 2	-0.27	0
Ifnl3	interferon lambda 3	-0.13	0
Il10	interleukin 10	n.d.	n.d.
Il11	interleukin 11	-0.2	0
Il12a	interleukin 12a	-0.02	0
Il12b	interleukin 12b	0.09	0
Il13	interleukin 13	0.07	0
Il15	interleukin 15	n.d.	n.d.
Il16	interleukin 16	0.2	0
Il17a	interleukin 17A	0.08	0
Il17b	interleukin 17B	0.02	0
Il17c	interleukin 17C	0.01	0
Il17d	interleukin 17D	0.04	0
Il17f	interleukin 17F	-0.18	0
Il18	interleukin 18	-0.21	0
Il19	interleukin 19	n.d.	n.d.
Il1a	interleukin 1 alpha	n.d.	n.d.
Il1b	interleukin 1 beta	-0.52	1
Il1f10	interleukin 1 family, member 10	n.d.	n.d.
Il1f5	interleukin 1 family, member 5 (delta)	-0.14	0
Il1f6	interleukin 1 family, member 6	n.d.	n.d.
Il1f8	interleukin 1 family, member 8	n.d.	n.d.
Il1f9	interleukin 1 family, member 9	n.d.	n.d.
Il2	interleukin 2	n.d.	n.d.
Il20	interleukin 20	n.d.	n.d.
Il21	interleukin 21	n.d.	n.d.
Il23a	interleukin 23, alpha subunit p19	0.66	1
Il24	interleukin 24	n.d.	n.d.
Il25	interleukin 25	0.05	0
Il27	interleukin 27	-0.12	1
Il3	interleukin 3	n.d.	n.d.
Il31	interleukin 31	-0.06	0
Il33	interleukin 33	n.d.	n.d.
Il34	interleukin 34	-0.2	0
Il4	interleukin 4	n.d.	n.d.
Il5	interleukin 5	n.d.	n.d.
Il6	interleukin 6	n.d.	n.d.
Il7	interleukin 7	n.d.	n.d.
Il9	interleukin 9	-0.08	0
Lif	leukemia inhibitory factor	-0.9	1

Lta	lymphotoxin A	-0.14	1
Ltb	lymphotoxin B	0.04	0
Mif	macrophage migration inhibitory factor	0.2	0
Osm	oncostatin M	0.31	1
Tgfb1	transforming growth factor, beta 1	-0.1	1
Tgfb2	transforming growth factor, beta 2	n.d.	n.d.
Tgfb3	transforming growth factor, beta 3	n.d.	n.d.
Tnf	tumor necrosis factor	0.76	3
Tnfsf10	tumor necrosis factor (ligand) superfamily, member 10	n.d.	n.d.
Tnfsf11	tumor necrosis factor (ligand) superfamily, member 11	-0.1	0
Tnfsf12	tumor necrosis factor (ligand) superfamily, member 12	n.d.	n.d.
Tnfsf13	tumor necrosis factor (ligand) superfamily, member 13	n.d.	n.d.
Tnfsf13b	tumor necrosis factor (ligand) superfamily, member 13b	0.26	0
Tnfsf14	tumor necrosis factor (ligand) superfamily, member 14	-0.02	0
Tnfsf15	tumor necrosis factor (ligand) superfamily, member 15	-0.28	0
Tnfsf18	tumor necrosis factor (ligand) superfamily, member 18	n.d.	n.d.
Tnfsf4	tumor necrosis factor (ligand) superfamily, member 4	n.d.	n.d.
Tnfsf8	tumor necrosis factor (ligand) superfamily, member 8	n.d.	n.d.
Tnfsf9	tumor necrosis factor (ligand) superfamily, member 9	0.51	1

* Group 0, all mRNAs with no significant change in expression levels during 2h stimulation of RAW264.7 macrophages with LPS; Group 1, mRNAs with a significant maximum at or after 1h; Group 2, mRNAs with a significant minimum at or after 1h; Group 3, mRNAs with a significant maximum before 1h; Group 4, mRNAs with a significant minimum before 1h.

Translationally up-regulated mRNAs are shaded in orange, translationally down-regulated mRNAs in blue.
