# Supplementary material for: Translational Regulation of Specific mRNAs Controls Feedback Inhibition and Survival during Macrophage Activation
Source: PLoS Genet. 2014 Jun 19;10(6):e1004368. doi: 10.1371/journal.pgen.1004368 (PMC4063670; doi:10.1371/journal.pgen.1004368)
Supplement: Table S4 — Feedback inhibitors and their expression features in 1 h LPS-stimulated RAW264.7 macrophages. mRNAs encoding feedback inhibitors of the TLR4 response are listed, together with the orthogonal distance (d) from the regression line in Figure 3 as a measure of their change in polysome association, and their expression pattern as determined by RNASeq in Figure 4. The list of feedback inhibitors was assembled by a systematic literature search. (PDF) [file pgen.1004368.s011.pdf]

Table S4 Feedback inhibitors and their expression features in 1 h LPS-stimulated RAW264.7 macrophages

Symbol	Name	Change of translation (d)	mRNA group*	PMID
Atf3	activating transcription factor 3	-0.27	3	19478204
Cd180	CD180 antigen	-0.08	0	19478204
Cdkn1a	cyclin-dependent kinase inhibitor 1A (P21)	0.47	1	19478204
Celf1	CUGBP, Elav-like family member 1	-0.16	1	21956941
Cited2	Cbp/p300-interacting transactivator, with Glu/Asp-rich carboxy-terminal domain, 2	0.1	1	21098220
Commd1	COMM domain containing 1	0.48	0	19233657
Cul2	cullin 2	-0.25	0	21772279
Cyld	cylindromatosis (turban tumor syndrome)	0.24	0	19233657
Dusp1	dual specificity phosphatase 1	0.68	3	22330073
Exosc9	exosome component 9	-0.15	0	21956941
Fbxw7	F-box and WD-40 domain protein 7	0.04	0	23575666
Hnrnpd	heterogeneous nuclear ribonucleoprotein D	-0.14	1	21956941
Ier3	immediate early response 3	0.97	3	19233657
Il1rl1	interleukin 1 receptor-like 1	0.08	0	19233657
Irak3	interleukin-1 receptor-associated kinase 3	-0.3	1	19233657
Itch	itchy, E3 ubiquitin protein ligase	-0.04	0	19233657
Khsrp	KH-type splicing regulatory protein	-0.49	0	21956941
Nfkbia	nuclear factor of kappa light polypeptide gene enhancer in B cells inhibitor, alpha	-0.21	3	19233657
Nfkbib	nuclear factor of kappa light polypeptide gene enhancer in B cells inhibitor, beta	-0.19	1	19233657
Nfkbid	nuclear factor of kappa light polypeptide gene enhancer in B cells inhibitor, delta	1,62	3	19233657
Nfkbie	nuclear factor of kappa light polypeptide gene enhancer in B cells inhibitor, epsilon	0	1	21772279
Nfkbiz	nuclear factor of kappa light polypeptide gene enhancer in B cells inhibitor, zeta	1.56	1	19233657
Nr4a1	nuclear receptor subfamily 4, group A, member 1	1.52	3	19325155
Otud7b	OTU domain containing 7B	-0.22	0	19233657
Pdlim2	PDZ and LIM domain 2	0.06	2	19233657
Pdpk1	3-phosphoinositide dependent protein kinase 1	-0.09	0	20584979
Pecam1	platelet/endothelial cell adhesion molecule 1	n,d,	n,d,	18025177
Pias1	protein inhibitor of activated STAT 1	-0.06	0	21772279
Pias4	protein inhibitor of activated STAT 4	-0.03	0	21772279
Ppp1ca	protein phosphatase 1, catalytic subunit, alpha isoform	0.26	0	19233657
Rc3h1	RING CCCH (C3H) domains 1	0.33	1	23663784
Rc3h2	ring finger and CCCH-type zinc finger domains 2	0	1	23663784
Sarm1	sterile alpha and HEAT/Armadillo motif containing 1	-0.16	0	21772279
Setd7	SET domain containing (lysine methyltransferase) 7	0	0	21772279
Sigirr	single immunoglobulin and toll-interleukin 1 receptor (TIR) domain	0.13	0	19233657
Slc39a8	solute carrier family 39 (metal ion transporter), member 8	0.12	0	23403290
Socs1	suppressor of cytokine signaling 1	-0.07	1	19233657
Socs3	suppressor of cytokine signaling 3	0.03	1	19233657
Tax1bp1	Tax1 (human T cell leukemia virus type I) binding protein 1	-0.09	0	19233657
Tnfaip3	tumor necrosis factor, alpha-induced protein 3	-0.13	3	19233657
Tnip1	TNFAIP3 interacting protein 1	-0.41	1	19233657
Tnip2	TNFAIP3 interacting protein 2	0.11	0	21772279
Tnip3	TNFAIP3 interacting protein 3	-0.36	1	19233657
Trib3	tribbles homolog 3 (Drosophila)	-0.25	1	19233657
Trim30a	tripartite motif-containing 30A	0.22	0	19233657

Trim38	tripartite motif-containing 38	n,d,	n,d,	22323536
Twist1	twist basic helix-loop-helix transcription factor 1	0.12	0	19233657
Twist2	twist basic helix-loop-helix transcription factor 2	0	0	19233657
Zc3h12a	zinc finger CCCH type containing 12A	0.64	1	21956941
Zc3h12d	zinc finger CCCH type containing 12D	-0.13	0	22036805
Zfp36	zinc finger protein 36	0.83	3	21956941
Zfp36l1	zinc finger protein 36, C3H type-like 1	0.46	3	21956941
Zfp36l2	zinc finger protein 36, C3H type-like 2	0.56	2	21956941

* Group 0, all mRNAs with no significant change in expression levels during 2h stimulation of RAW264.7 macrophages with LPS; Group 1, mRNAs with a significant maximum at or after 1h; Group 2, mRNAs with a significant minimum at or after 1h; Group 3, mRNAs with a significant maximum before 1h; Group 4, mRNAs with a significant minimum before 1h.
